# Characterization of Anti-Poliovirus Compounds Isolated from Edible Plants

**DOI:** 10.3390/v15040903

**Published:** 2023-03-31

**Authors:** Minetaro Arita, Hiroyuki Fuchino

**Affiliations:** 1 Department of Virology II, National Institute of Infectious Diseases, 4-7-1 Gakuen, Musashimurayama-shi 208-0011, Tokyo, Japan; 2 Research Center for Medicinal Plant Resources, National Institutes of Biomedical Innovation, Health and Nutrition, 1-2 Hachimandai, Tsukuba 305-0843, Ibaraki, Japan

**Keywords:** virus, picornavirus, enterovirus, antiviral, edible plant, PI4KB

## Abstract

Poliovirus (PV) is the causative agent of poliomyelitis and is a target of the global eradication programs of the World Health Organization (WHO). After eradication of type 2 and 3 wild-type PVs, vaccine-derived PV remains a substantial threat against the eradication as well as type 1 wild-type PV. Antivirals could serve as an effective means to suppress the outbreak; however, no anti-PV drugs have been approved at present. Here, we screened for effective anti-PV compounds in a library of edible plant extracts (a total of 6032 extracts). We found anti-PV activity in the extracts of seven different plant species. We isolated chrysophanol and vanicoside B (VCB) as the identities of the anti-PV activities of the extracts of *Rheum rhaponticum* and *Fallopia sachalinensis*, respectively. VCB targeted the host PI4KB/OSBP pathway for its anti-PV activity (EC_50_ = 9.2 μM) with an inhibitory effect on in vitro PI4KB activity (IC_50_ = 5.0 μM). This work offers new insights into the anti-PV activity in edible plants that may serve as potent antivirals for PV infection.

## 1. Introduction

Poliovirus (PV) is a small non-enveloped virus with a positive-sense single-stranded RNA genome of about 7500 nt belonging to the family *Picornaviridae*, including poliovirus (PV, species *Enterovirus C*) [1]. PV is the causative agent of poliomyelitis, which mainly affects children under 5 years of age, and is a target of global eradication by the World Health Organization (WHO). Through vaccination programs of the Global Polio Eradication Initiative beginning in 1988 with a live oral PV vaccine (OPV) and/or an inactivated PV vaccine (IPV), type 2 and 3 wild-type PVs (WPVs) have been eradicated (declared in 2015 and 2019, respectively), and only Pakistan and Afghanistan remain as endemic countries of type 1 WPV as of 2022. However, circulating vaccine-derived PV (cVDPV) remains a substantial threat against the eradication (724 cases in 2022), especially type 2 cVDPV that emerged after the global cessation of type 2 OPV in 2016 [2], as well as type 1 WPV (30 cases in 2022) [3]. Transmission of PV could be re-established by the importation of the following strains: polio cases by type 1 WPV in Malawai in 2021 and Mozambique in 2022, a case by type 2 cVDPV, and the silent circulation in the United States of America and [4] the United Kingdom in 2022 [5]. To interrupt PV circulation, only an OPV campaign for a potentially susceptible population in the area could serve as the effective mean at present (in case of an outbreak response in Israel, the target population was children under 10 years of age who have received at least one dose of IPV) [6]. In addition to conventional OPV, novel OPV type 2 (nOPV2), which was designed to have more genetic stability than type 2 OPV and to decrease the risk of VDPV [7], is available for the outbreak response under Emergency Use Listing of the WHO authorized in 2020. Currently, nOPV2 has mainly been used in African countries since 2021 (reviewed in [8]).

In the global eradication, antivirals for PV are anticipated to serve as an effective means to suppress cVDPV outbreaks and to treat patients chronically infected with PV [9,10]. As the candidate compounds, direct-acting antivirals targeting viral capsid proteins (inhibitors of viral binding/uncoating steps), proteases (viral 2A and 3C/3CD proteins), helicase (viral 2C protein), and polymerase (viral 3D protein) have been reported (reviewed in [11]). Host-targeting antivirals have also been reported, including inhibitors for host GBF1 (a guanine-nucleotide exchange factor) [12,13], eIF4A (protein synthesis) [14], HSP90 (folding of viral capsid proteins) [15], DHODH (de novo pyrimidine synthesis) [16,17,18], ribosome (protein synthesis) [19], PI4KB (phosphatidylinositol 4-monophosphate production) [20,21,22,23,24,25,26,27], and OSBP (exchanger of cholesterol and phosphatidylinositol 4-monophosphate) [28,29,30,31,32]. However, there is no antiviral available for PV infection at present.

Target population of PV is mainly for children under five years of age as well as other enteroviruses (EVs) [33,34]; therefore, safety is one of the major challenges for the antiviral development [10]. In a previous study, a highly active anti-EV compound (EC_50_ = 2.0 μM) was isolated from avocado [35], suggesting that edible plants provide a promising source for potent anti-EV compounds. Here, we isolated anti-PV compounds from edible plants, *Rheum rhaponticum* and *Fallopia sachalinensis*, and analyzed the potency and the mechanism of action of their isolated compounds.

## 2. Materials and Methods

Cells. Cells were cultured as monolayers in Dulbecco’s modified Eagle medium (DMEM, FUJIFILM Wako Pure Chemical Corporation, Osaka, JPN, 044-29765) supplemented with 10% foetal calf serum (FCS). RD cells (human rhabdomyosarcoma cells) were used for the titration of PV and evaluation of anti-PV activity of plant extracts. HEK293 cells (human embryonic kidney cells) were used for the production of type 1 PV pseudovirus (PV1_pv_). A *PI4KB*-knockout RD cell line (RD[Δ*PI4KB*]) was used to evaluate the antiviral effects of plant extracts targeting PI4KB/OSBP-independent viral replication [36].

Viruses. Type 1 PV Sabin 1 strain (PV1[Sabin 1]) (GenBank: AY184219), type 3 PV Sabin 3 strain (PV3[Sabin 3]) (GenBank: AY184221), EV-A71 (Nagoya) (GenBank: AB482183), and EV-D68 (Fermon) (GenBank: AY426531) were used for the screening of plant extracts. Luciferase-encoding Sendai virus (SeV-luc) was a kind gift from Atsushi Kato. PV1_pv_ mutants were produced with a firefly luciferase-encoding type 1 PV Mahoney strain (PV1[Mahoney]) (GenBank: V01149) replicon and the capsid proteins of PV1(Mahoney) [37]. The PV1_pv_ mutants used in this study are as follows: an enviroxime (PI4KB inhibitor)-resistant mutant (with a G5318A [3A-Ala70Thr] mutation)[21], a guanidine hydrochloride (GuaHCl) (viral 2C helicase inhibitor)-resistant mutant (with a U4614A [2C-Phe164Tyr] mutation) [38], a brefeldin A (GBF1 inhibitor)-resistant mutant (with a G4361A [2C-Val80Ile] mutation and a C5190U [3A-Ala27Val] mutation) [39], a rupintrivir (viral 3C protease inhibitor)-resistant mutant (with a G5819A [3C-Gly128Ser] mutation) [40], a disoxaril (viral capsid-binding uncoating inhibitor)-resistant mutant (with an A3059U [VP1-Ile194Phe] mutation)[41], the Δ*PI4KB*-resistant [−2C] mutant (a PI4KB/OSBP-independent mutant) (with a U3623C [2A-Phe80Leu] mutation, a U3881C [2B-Phe17Leu] mutation, a G3892U [2B-Gln20His] mutation, an A5269U [3A-Glu53Asp] mutation, and an A5270U [3A-Arg54Trp] mutation) [36]. Plasmids for the rupintrivir-resistant mutant and the disoxaril-resistant mutant were constructed in this study as below.

Chemicals. Chrysophanol and vanicoside B (VCB) were obtained from the roots of *R. rhaponticum* and of *F. sachalinensis*, respectively (purity >95% determined by NMR, HPLC). MDL-860 was a kind gift from Angel S. Galabov (purity >99.5%, determined by NMR) [42].

General methods for molecular cloning. *Escherichia* *coli* strain XL10gold (Agilent Technologies, Inc., Santa Clara, CA, USA) was used for the preparation of plasmids. PCR was performed using KOD Plus DNA polymerase (TOYOBO CO., LTD., Osaka, Japan). DNA sequencing was performed using a BigDye Terminator v3.1 cycle sequencing ready reaction kit (Thermo Fisher Scientific Inc., Waltham, MA, USA) and then analyzed with a 3500xL genetic analyzer (Thermo Fisher Scientific Inc., Waltham, MA, USA).

Plasmids:

Rupintrivir-resistant PV replicon. A resistant mutation to rupintrivir was introduced into a plasmid encoding the cDNA of a PV replicon (pPV-Fluc mc) [43], by PCR with primer set 1.

Primer set 1:

5′-GGATATCTAAATCTCAGTGGGCGCCAAAC-3′

5′-GTTTGGCGCCCACTGAGATTTAGATATCC-3′

Disoxaril-resistant PV capsid expression vector. A resistant mutation to disoxaril was introduced into a plasmid expression vector for the capsid protein of PV1(Mahoney) [37], by PCR with primer set 2.

Primer set 2:

5′-CAGCTCCAGCCCGGTTCTCGGTACCGTATG-3′

5′-CATACGGTACCGAGAACCGGGCTGGAGCTG-3′

Edible plant extract library. A plant extract library was prepared in the Research Center for Medicinal Plant Resources (NIBIOHN), by the methanol extraction of dried and pulverized plant materials, with evaporation, dissolution in DMSO, and filtration. The concentration of the final DMSO solution was adjusted to 40 mg/mL for all extracts. This plant extract library is a collection of extracts from a wide range of wild plants in Japan, which can be widely used not only in drug discovery but also in the life sciences field, such as health food development. All the original plants in the library are annotated with information on whether or not they have been eaten by humans. The presence of food experience in the original plant reflects the safety of the extract for human beings, which is useful information for drug discovery. We selected samples from this library derived from the original plants with food experience and used them in this study.

Screening for anti-PV compounds from edible plant extract library. RD cells (2.0 × 10^4^ cells per well in 50 μL medium) were cultured in 96-well plates, and then infected with PV1(Sabin 1) (2000 50% cell culture infectious dose [CCID_50_]) in the presence of a plant extract (final 0.2 mg/mL) (total 200 μL/well). The cells were incubated at 37 °C, and then observed for CPE at 1, 2, 3, and 7 days post-infection (p.i.). 

Measurement of cytotoxicity and anti-PV activity of compounds. For the measurement of cytotoxicity, RD cells (8 × 10^3^ cells per well in 20 μL medium) were cultured at 37 °C in 384-well plates (781,080, Greiner Bio-One, Kremsmünster, Austria), followed by the addition of 20 μL of a compound solution at an indicated final concentration. The cells were incubated at 37 °C for 7 h or 2 days and then the cell viability was measured by using a CellTiter-Glo 2.0 Cell Viability Assay kit (G9241, Promega Corporation, Madison, WI, USA) using a 2030 ARVO X luminometer (PerkinElmer, Waltham, MA, USA). The 50% cytotoxic concentration (CC_50_) values were determined by a nonlinear regression analysis of the dose–response curves.

For the measurement of anti-PV activity with PV1_pv_, RD cells (8 × 10^3^ cells per well in 20 μL of medium) in 384-well plates (781,080, Greiner Bio-One, Kremsmünster, Austria) were inoculated with 10 μL of PV1_pv_ (800 infectious units [IU]) and 10 μL of compound solution at an indicated final concentration. The cells were incubated at 37 °C for 7 h. Luciferase activity in the infected cells was measured at 7 h p.i. with the Steady-Glo luciferase assay system (Promega Corporation, Madison, WI, USA) using a 2030 ARVO X luminometer (PerkinElmer, Waltham, MA, USA). The 50% effective concentration (EC_50_) values were determined via a nonlinear regression analysis of the dose–response curves. 

For the measurement of anti-PV activity of VCB, RD cells (2.8 × 10^4^ cells per well in 50 μL of medium) were cultured in 96-well plates, and then infected with PV1(Sabin 1) (100 CCID_50_), EV-A71(Nagoya) (1000 CCID_50_), and EV-D68(Fermon) (10^4^ CCID_50_) in the presence of VCB at an indicated final concentration (total 200 μL/well). The cells were incubated at 37 °C for 2 days (for PV), 35 °C for 4 days (for EV-A71), or 33 °C for 4 days (for EV-D68). The cells were then fixed and stained with formalin and crystal violet (final concentration of 5% and 0.25%, respectively).

Measurement of inhibitory effect of compounds on in vitro PI4KB activity. The in vitro activity of purified GST-PI4KB (PV5277, Thermo Fisher Scientific Inc., Waltham, MA, USA) was evaluated by using an ADP-Glo Lipid Kinase Systems kit (Promega Corporation, Madison, WI, USA) as previously described. In a total 5.5 µL reaction solution, the PI4KB activity of 32 ng of purified GST-PI4KB (final concentration of 48 nM) with lipid substrates (0.025 mg/mL of phosphatidylinositol and 0.075 mg/mL or phosphatidylserine) and 25 µM of ATP was measured in the presence or the absence of compounds. The net signals of the mock-treated samples were taken as 100% of the PI4KB activity. The 50% inhibitory concentration (IC_50_) values of the compounds were determined by nonlinear regression analyses of the dose–response curves.

**Statistical analysis**. The results of the experiments are shown as means with standard deviations. Values of *p* < 0.05 by one-tailed *t* tests were considered to indicate a significant difference, and were indicated by asterisks (* *p* < 0.05, ** *p* < 0.01, *** *p* < 0.001).

## 3. Results

### 3.1. Screening of Edible Plant Extracts for Anti-PV Activity

**Figure 1:**
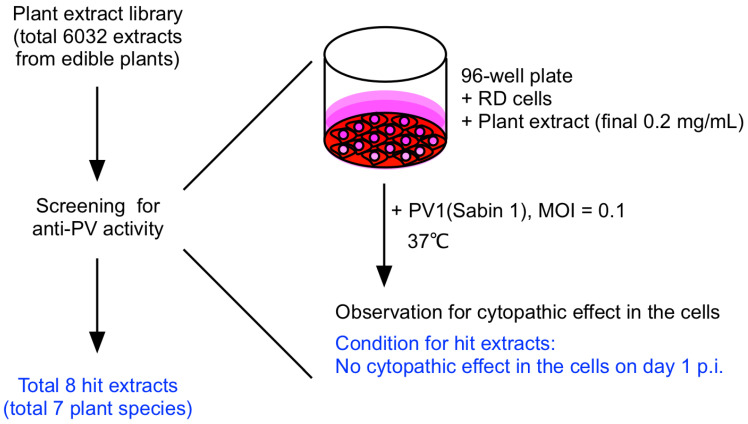
Summary of the screening of anti-PV compound from edible plant extract library.

We screened a total of 6032 edible plant extracts for anti-PV activity in RD cells as that previously performed for the screening for anti-EV-D68 activity [35] (Figure 1). The plant extracts were added to the RD cells (final concentration of 0.2 mg/mL), and then infected with PV1(Sabin 1) at a multiplicity of infection (MOI) of 0.1. The extracts that completely protected the cells from the viral infection after 1 day post-infection (p.i.) were identified as initial hit extracts. We identified 8 hit extracts, which consisted of 7 plant species. In this study, we focused on the identification of the identity of the antiviral effects in *R*. *rhaponticum* and *F. sachalinensis,* among the hits for availability of the plant materials.

### 3.2. Purification and Structure Determination of Anti-PV Compound in R. Rhaponticum

**Figure 2:**
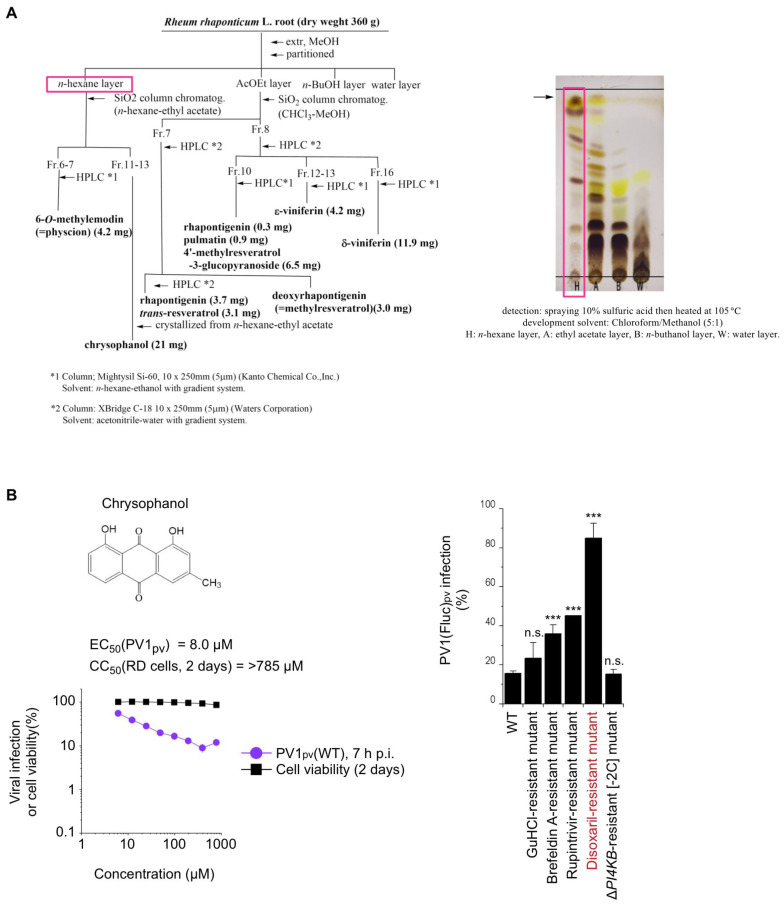
Identification and purification of an anti-PV compound from *R. rhaponticum* extract. (**A**) Procedure of purification of anti-PV compound from *R. rhaponticum* extract. Boxes highlight the fraction containing chrysophanol. Arrow indicates the band of chrysophanol. (**B**) Antiviral effect of chrysophanol on PV infection. (Left) Structure, cytotoxicity, and antiviral effect of chrysophanol. Viral infection and viability of RD cells in the presence of chrysophanol are shown. RD cells were infected with PV1_pv_, then luciferase activity was measured at 7 h p.i. Marked precipitation of chrysophanol was observed above 98 μM. Viral infection or the cell viability in the absence of chrysophanol were taken as 100%. (Right) Inhibitory effect of chrysophanol on the infection of a panel of drug-resistant PV1_pv_ mutants. PV1_pv_ infection at 7 h p.i. in RD(WT) cells in the presence or absence of chrysophanol (790 μM) is shown. PV1_pv_ infection in the absence of chrysophanol is taken as 100%. n.s., not significant. ***, *p* < 0.001.

Methanolic extracts of *R. rhaponticum* (*Polygonaceae*) root was partitioned with *n*-hexane, ethyl acetate, *n*-buthanol, and water, successively. The *n*-hexane layer was purified via silica-gel column chromatography, then, HPLC to give chrysophanol (21 mg) [44], and 6-*O*-methylemodin (4.2 mg) [45] (Figure 2). The ethyl acetate layer was separated by silica-gel column chromatography and HPLC (Supplementary data 1), repeatedly, to obtain rhapontigenin (3.7 mg) [46], *trans*-resveratrol (3.1 mg) [47], pulmatin (0.9 mg) [48], 4-methylresveratrol-3-glucopyranoside (6.5 mg) [49], ε-viniferin (4.2 mg) [50], δ-viniferin (11.9 mg) [51], and deoxyrhapontigenin (3.0 mg) [46]. Their chemical structures were determined by NMR and LC/MS (Supplementary data 2). The major active component in the active fractions was revealed to be chrysophanol (Figure 2A). Chrysophanol has been reported as the anti-PV component of an Australian medicinal plant *Dianella longifolia,* and targets the early stage of PV infection [52]. The antiviral effect of chrysophanol was specific to PV; no antiviral effect was observed on the infection of EV-A71 or EV-D68 (Figure S1). The EC_50_ of chrysophanol for type 1 PV pseudovirus (PV1_pv_) infection was 8.0 μM; however, the infection could be suppressed only moderately even at 790 μM (12% of that in mock-treated cells), (Figure 2B). Consistent with a previous report, a disoxaril (viral capsid-binding uncoating inhibitor)-resistant mutant showed substantial resistance to purified chrysophanol (Figure 2B). This suggested that chrysophanol was the major identity for the anti-PV activity of the *Rheum rhaponticum* extract and targeted the PV capsid protein.

### 3.3. Purification and Structure Determination of Anti-PV Compound in F. Sachalinensis 

**Figure 3:**
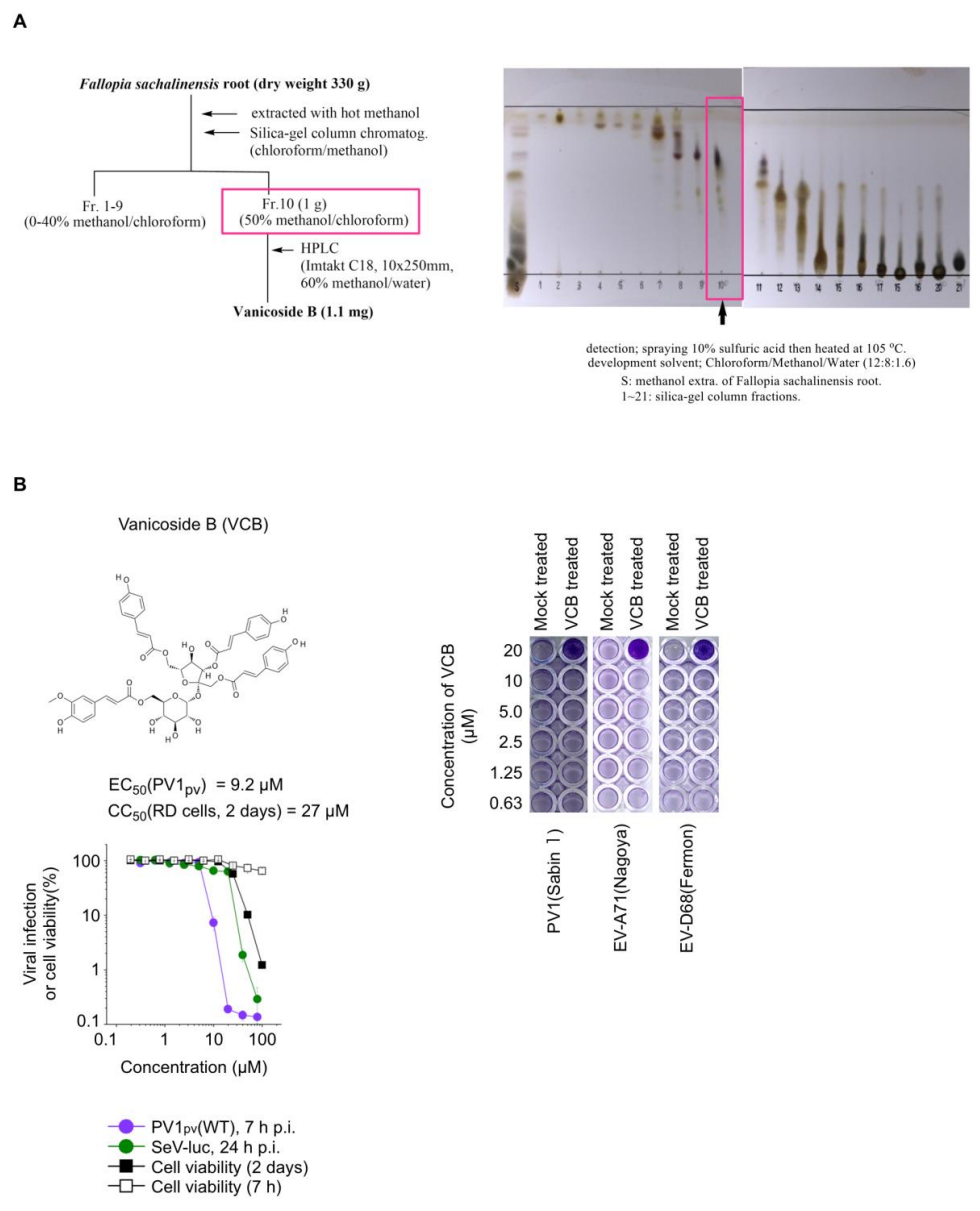
Identification and purification of an anti-PV compound from *F. sachalinensis* extract. (**A**) Procedure of purification of anti-PV compound from *F. sachalinensis* root extract. Boxes highlight the fraction containing vanicoside B. (**B**) Antiviral effect of VCB on PV infection. (Left) Structure of VCB, cytotoxicity, and antiviral activity of VCB. Viral infection and viability of RD cells in the presence of VCB are shown. RD cells were infected with PV1_pv_ or SeV-luc, then luciferase activity was measured at 7 h p.i. (for PV1_pv_) or 24 h p.i. (for SeV-luc). Viral infection or the cell viability in the absence of VCB were taken as 100%. (Right) Antiviral effect of VCB on EV infection. RD cells on 96-well plates were infected with EV at an MOI of 0.005 (for PV), 0.05 (for EV-A71) or 0.5 (for EV-D68) in the absence (mock-treated) or the presence (VCB treated) of VCB at the indicated final concentration. The cells were fixed and stained at 2 days p.i. (for PV) or 4 days p.i. (for EV-A71 and EV-D68). The data are representative of two independent experiments with two to three biological replicates.

The methanolic extracts of the *F. sachalinensis* (*Polygonaceae*) root was purified by silica-gel column chromatography with chloroform–methanol as an eluent to give 20 fractions (Figure 3A). Fractions eluted with 50% methanol/chloroform were combined and subjected to HPLC separation to obtain vanicoside B (VCB, 1.1 mg). The chemical structure was determined via comparison with NMR data from the literature [53].

### 3.4. Characterization of Anti-PV Activity of VCB

To evaluate the potency of VCB as an anti-PV compound, we determined the 50% cytotoxic concentration (CC_50_) in human RD cells and 50% effective concentration (EC_50_) for the PV infection of VCB (Figure 3B). The cytotoxicity of VCB was not observed when the cells were treated with 100 μM VCB for 7 h, but the CC_50_ of VCB after 2 days of treatment was 27 μM. The EC_50_ of VCB for PV1_pv_ infection was 9.2 μM. The selectivity index (SI) of VCB for anti-PV activity in RD cells was 2.9, suggesting a low specificity for the anti-PV activity of VCB (e.g., SI of PI4KB inhibitors for the anti-PV activities could be >1000) [25]. VCB protected the RD cells from the infection of PV1(Sabin 1), EV-A71(Nagoya), or EV-D68(Fermon) only at 20 μM, suggesting that the potential therapeutic window of VCB is quite narrow.

### 3.5. Mechanism of Anti-PV Activity of VCB

**Figure 4:**
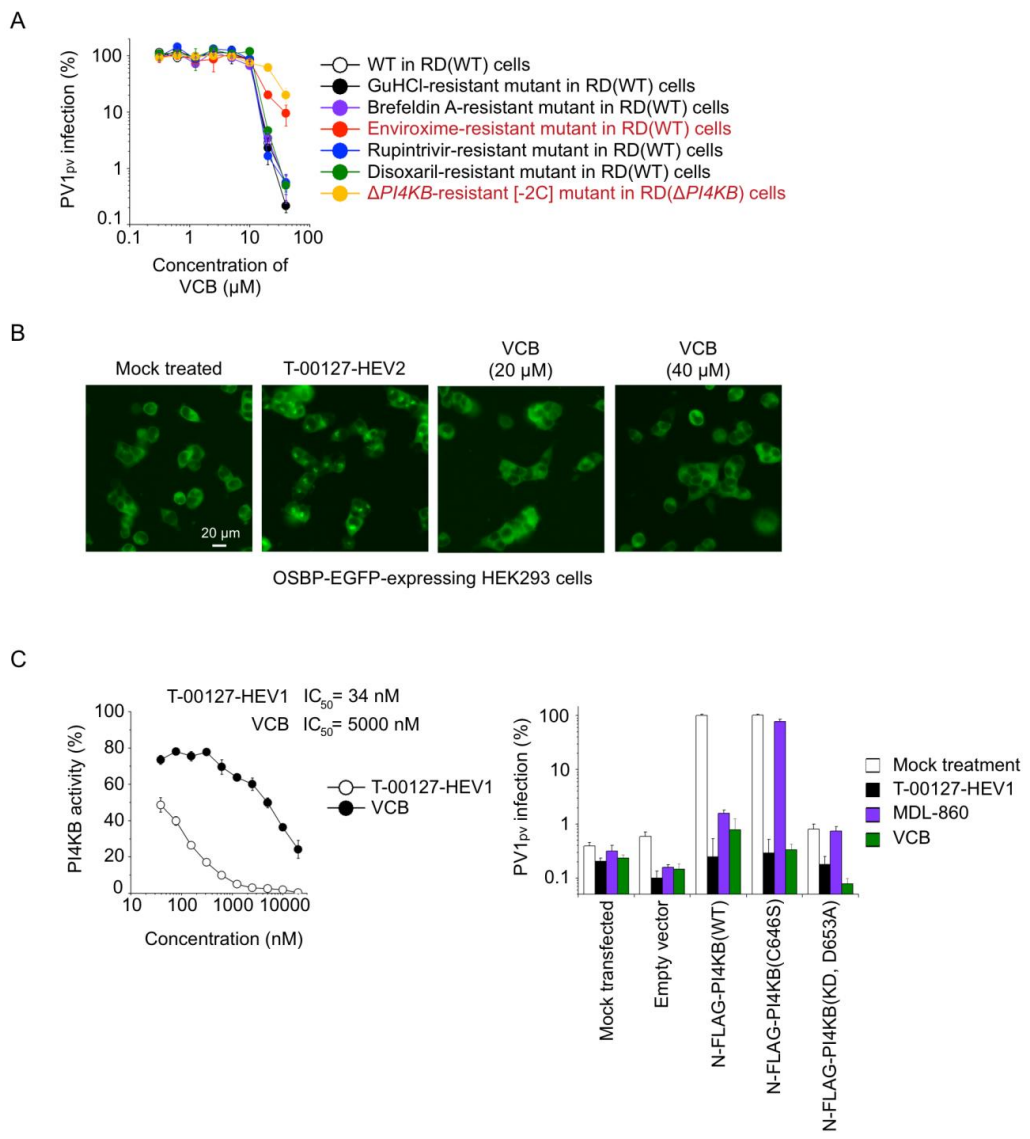
VCB is a novel PI4KB inhibitor. (**A**) Inhibitory effect of VCB on the infection of a panel of drug-resistant PV1_pv_ mutants. PV1_pv_ infection at 7 h p.i. in RD(WT) cells in the presence or absence of VCB is shown, except for the infection of the Δ*PI4KB*-resistant [−2C] mutant, which was analyzed at 17 h p.i. in RD(Δ*PI4KB*) cells. PV1_pv_ infection in the absence of VCB is taken as 100%. (**B**) OSBP-EGFP-expressing HEK293 cells were incubated at 37 °C for 30 min in the presence of the compounds (10 μM T-00127-HEV2, 20 or 40 μM VCB, respectively). Localization of OSBP-EGFP in the cells is shown. (**C**) (Left) Inhibitory effect of VCB on in vitro PI4KB activity. (Right) *Trans*-rescue of PV infection in RD(Δ*PI4KB*) cells by indicated PI4KB variants in the presence of the compounds (20 μM T-00127-HEV1, 40 μM MDL-860, or 20 μM VCB, respectively). PV1_pv_ infection in RD(Δ*PI4KB*) cells transfected with pTK-N-FLAG-PI4KB(WT, C646S, or D653A variants) in the absence of the compound is taken as 100%. The data are representative of two independent experiments with three biological replicates.

To evaluate the specificity of the anti-PV activity for VCB, we analyzed the antiviral activity with a panel of drug-resistant PV mutants in parental (wild-type) RD cells (RD[WT]) and *PI4KB*-knockout RD cells (RD[Δ*PI4KB*]) (Figure 4A). The panel included resistant mutants to the direct-acting antivirals guanidine hydrochloride (GuHCl) (viral 2C helicase inhibitor), rupintrivir (viral 3C protease inhibitor), and disoxaril (viral capsid-binding uncoating inhibitor) and to the host-targeting antivirals brefeldin A (host GBF1 inhibitor), enviroxime (host PI4KB inhibitor), and a PI4KB/OSBP-independent mutant (Δ*PI4KB*-resistant [−2C]). The antiviral effect of VCB on the infection of the PV1_pv_ mutants was analyzed in RD(WT) cells, except for that of the Δ*PI4KB*-resistant (−2C) mutant. The potential antiviral effects on PI4KB/OSBP-independent replication was analyzed in the infection of the Δ*PI4KB*-resistant (−2C) mutant in RD(Δ*PI4KB*) cells. Among the mutants, the enviroxime-resistant mutant and Δ*PI4KB*-resistant (−2C) mutant showed significant resistance to VCB, suggesting that VCB targets the host PI4KB/OSBP pathway in PV replication.

To further dissect the target of VCB, we analyzed the effect of VCB on the subcellular localization of host OSBP. OSBP relocalizes to the Golgi in the presence of OSBP inhibitors via the lipid-transfer domain [30,54,55]. HEK293 cells overexpressing C-terminally EGFP-fused OSBP were treated with an OSBP inhibitor T-00127-HEV2 or VCB (Figure 4B). While treatment of the cells with T-00127-HEV2 caused the relocalization of the ectopically expressed OSBP to the Golgi, the treatment with VCB did not affect the subcellular localization, suggesting that OSBP is not the target of VCB. Next, we analyzed the effect of VCB on the PI4KB activity (Figure 4C). VCB showed an inhibitory effect on the in vitro PI4KB activity albeit with low potency (IC_50_ = 5000 nM) compared to a PI4KB inhibitor T-00127-HEV1 (IC_50_ = 34 nM), which possibly targets the ATP-binding site of PI4KB, similar to its analogue [26]. We also analyzed a potential allosteric effect of VCB on the PI4KB activity. VCB inhibited the activity of a PI4KB variant (C646S) as well as T-00127-HEV1, in contrast to MDL-860 that has an allosteric inhibitory effect on PI4KB via a covalent modification of the Cys646 residue [27,56]. These results suggested that PI4KB is the direct target of VCB for the anti-PV activity.

## 4. Discussion

Several potent anti-EV compounds have been isolated from plants: pachypodol (Ro 09-0179) (PI4KB inhibitor) [57], oxoglaucine (PI4KB inhibitor) [58,59], chrysin (viral 3C protease inhibitor) [60], prunin (viral protein synthesis inhibitor) [61], and avoenin (viral capsid-binding uncoating inhibitor) [35]. However, the availability of these plant-derived compounds for treatment or prophylactic use in PV infection has yet to be established. In the present study, we isolated chrysophanol and vanicoside B (VCB) as anti-PV compounds from an *R. rhaponticum* extract and *F. sachalinensis* extract, respectively. 

The petiole of *R. rhaponticum* is edible, called rhubarb, and is used mainly as a jam. On the other hand, the roots of many *Rheum* spp. are considered medicinal and laxative because they contain many anthraquinones. *F. sachalinensis* is a large herbaceous plant whose grass can grow up to 2 m tall. The Ainu tribe of Japan traditionally eats its young stems and sprouts. The rhizome of *F. sachalinensis* has antibacterial, antitussive, and diuretic properties, as well as an improvement in its laxative effects. The Japanese name for this plant (“ooitadori”) is derived from the fact that when bruised, its leaves can be applied to the affected area to relief pain. Approximately 360 g of the root of *R. rhaponticum* contained 0.2 g of chrysophanol (about 0.05%*w*/*w*). The content of VCB in the roots of *F. sachalinensis* dry was estimated to be about 0.001%*w*/*w*, based on the weight of the isolate and its presence in other fractions. Although the roots of these two plant species are not edible parts, other parts have been traditionally consumed. Therefore, a certain degree of safety is considered to be assured.

Chrysophanol is a well-known purgative component of the roots of the *Rheum* spp. and Senna leaf (the leaves of *Cassia angustifolia*, *C. acutifolia*). It has also been reported as a constituent of *Fallopia japonica*, a closely related plant to *F. sachalinensis*. Chrysophanol is also known as an anti-PV compound [52]. Emodin, a compound structurally related to chrysophanol, has anti-EV activity targeting viral protein synthesis or virion maturation [62,63]. Chrysophanol targeted a site of a PV capsid protein similar to the capsid-binding uncoating inhibitors; however, the inhibitory effect of the chrysophanol on PV1_pv_ infection was rather weak even at a high concentration (about an 8-fold reduction at 790 μM, Figure 2B). The infection cycle of PV1_pv_ includes viral binding, uncoating, and replication, but not virion production (assembly, encapsidation, maturation, and egress) [37]; therefore, chrysophanol may have additional targets after the replication step, such as virion maturation, similar to emodin. The partial resistance of a brefeldin A-resistant mutant and a rupintrivir-resistant mutant against chrysophanol suggest the effects on the replication step that could have functional coupling to virion production [64,65]. Pocapavir (viral capsid-binding uncoating inhibitor) [66,67] and V-7404 (3C protease inhibitor) [68,69,70] have been considered as candidate antivirals in the polio eradication program [71,72]. The availability of chrysophanol in a broad plant species would allow further evaluation of the potency of the extracts, possibly in combination with other drugs/extracts with different antiviral mechanisms.

VCB was first isolated from nature in 1994 as a protein kinase C inhibitor from *Polygonum pensylvanicum* (*Polygonaceae*) together with vanicoside A [73]. Vanicosides have inhibitory effects on the viral proteases of the human immunodeficiency virus or SARS-CoV-2 [74,75], but its antiviral effects have yet to be evaluated. Unexpectedly, resistant mutants (the enviroxime-resistant mutant and Δ*PI4KB*-resistant [−2C] mutant) suggested that the target of VCB for the anti-PV activity is the host PI4KB/OSBP pathway rather than viral proteases (Figure 3). The inhibitory effect on in vitro PI4KB activity (IC_50_ = 5.0 μM) suggested that VCB is a novel PI4KB inhibitor. PI4KB is a host factor required for the replication of EV identified by Hsu et al. [22]. The subsequent analysis on a group of anti-EV compounds (designated enviroxime-like compounds), which have PI4KB and an unknown factor as the targets for the anti-EV activity [23,29], revealed the host oxysterol-binding protein (OSBP) family I (OSBP and OSBP2/ORP4) as another target of this compound group [30,31]. PI4KB and OSBP form an inseparable functional axis for the formation of a viral replication complex by providing phosphatidylinositol 4-monophosphate (PI4P) for the recruitment of OSBP on viral replication organelles (ROs), and the accumulation of unesterified cholesterol on the ROs by OSBP [76]. This process enhances the cleavage of the viral 3AB protein and development of the RO for the synthesis of viral plus-strand RNA [36,57,77,78,79]. In addition to the 3AB protein, the viral 2B protein is essential to complement the functional axis [36,65], while the functional role of the 2B protein remains largely unknown. PI4KB inhibitors generally show low cytotoxicity to cultured cells [20,21,23,28,43,80,81]; however, the antiproliferative effect in lymphocytes [80] and lethality in a mouse line [81] raised concerns on the safety in vivo. A recent study revealed a protective effect in PI4KB heterozygous kinase-dead mice against EV infection and the therapeutic potency of a specific PI4KB inhibitor in vivo [82], supporting the potential safety of PI4KB inhibitors in clinical use as opposed to earlier findings. Therefore, the PI4KB inhibitors isolated from edible plants could have a more important role than ever thought.

The limitations of this study include elucidation of the mechanism of the inhibitory effect of VCB on PI4KB activity and its off-target effect against clinical applicability. Most of the identified PI4KB inhibitors target the ATP-binding site of PI4KB [26,83], with MDL-860 as the exception. The inhibitory effect of VCB on in vitro PI4KB activity might suggest the direct interaction with PI4KB (Figure 3C), but the target site remained to be determined. While the specificity to the PI4KB/OSBP pathway in terms of the anti-PV activity was clear, we could not exclude the potential contribution of the off-target effect of VCB (CC_50_ = 27 μM) to the observed anti-PV activity, which had quite a narrow therapeutic window (complete protection of the cells from PV1[Sabin 1] infection at 20 μM).

## Data Availability

Raw data sets not included in this paper are available from the corresponding authors upon request.

## Supplementary Materials

The following supporting information can be downloaded at: https://www.mdpi.com/article/10.3390/v15040903/s1, Figure S1: Antiviral effect of chrysophanol on EV infection. Supplementary data 1: HPLC chart. Supplementary data 2: NMR spectra.

## List of Abbreviations

CCID_50_	50% cell culture infectious dose
cVDPV	circulating vaccine-derived poliovirus
EC_50_	50% effective concentration
EV	enterovirus
GuHCl	guanidine hydrochloride
IC_50_	50% inhibitory concentration
IPV	inactivated PV vaccine
MOI	multiplicity of infection
OPV	oral PV vaccine
OSBP	oxysterol-binding protein
P.i.	post-infection
PI4P	phosphatidylinositol 4-monophosphate
PV	poliovirus
PV1_pv_	type 1 PV pseudovirus
RO	replication organelle
SeV-luc	luciferase-encoding Sendai virus
SI	selectivity index
VCB	vanicoside B
