# Supplementary figures and images for: Characterization of Anti-Poliovirus Compounds Isolated from Edible Plants
Source: Viruses. 2023 Mar 31;15(4):903. doi: 10.3390/v15040903 (PMC10145814; doi:10.3390/v15040903)

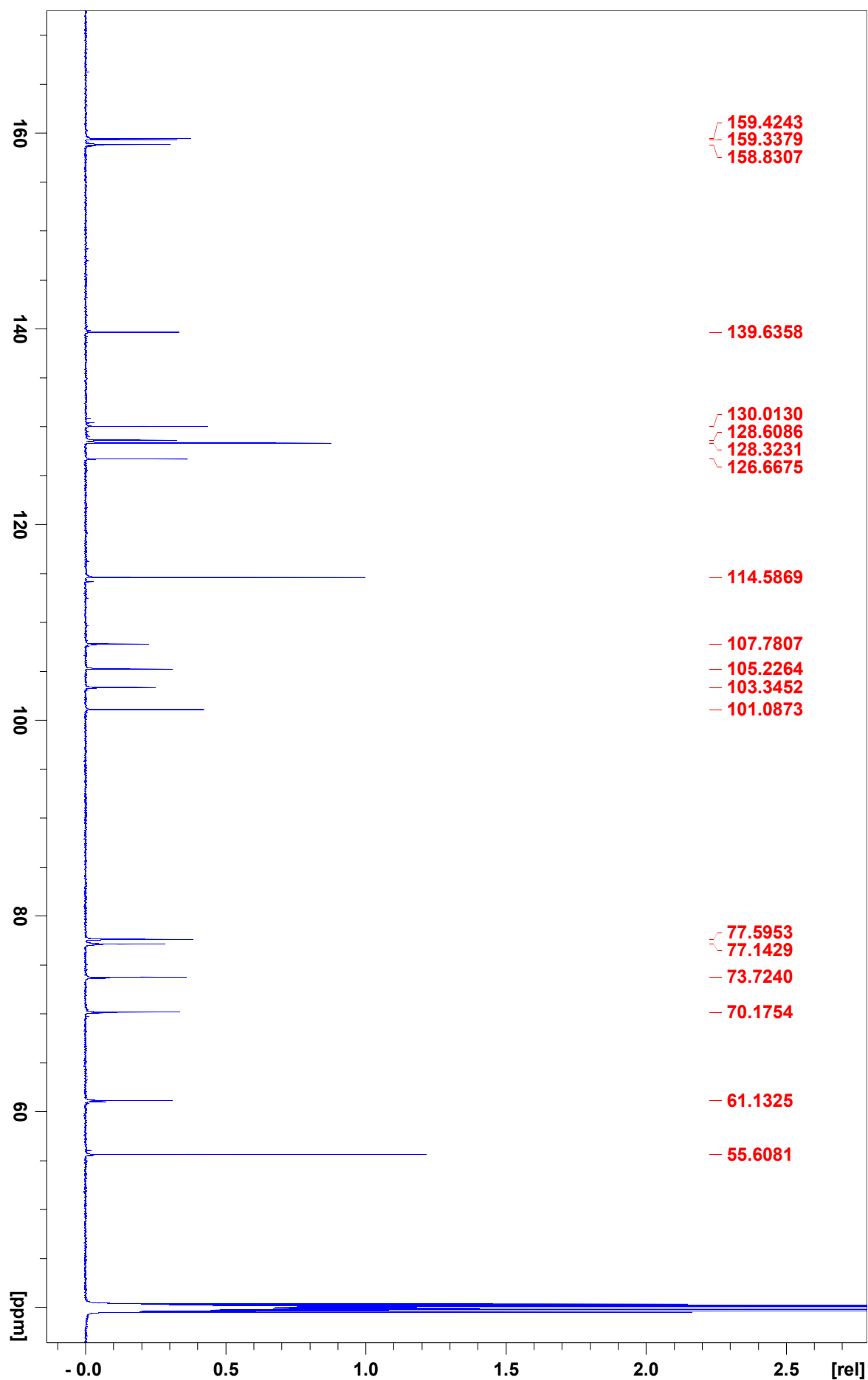

Supplement: Supplementary file 1 [file viruses-15-00903-s001.zip › 4'-methylressveratrol-3-glucoside(13C).pdf]

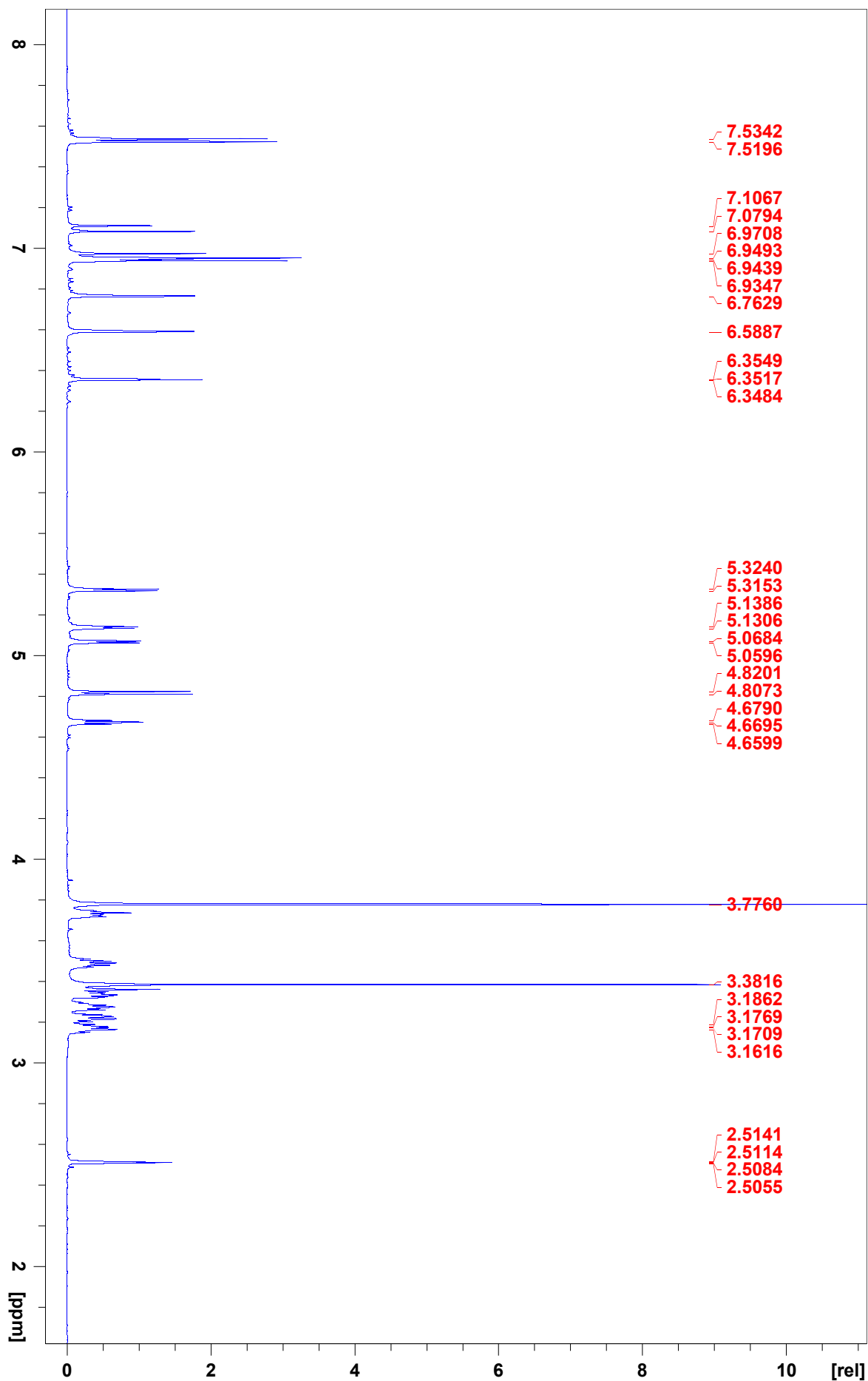

Supplement: Supplementary file 1 [file viruses-15-00903-s001.zip › 4'-methylressveratrol-3-glucoside(1H).pdf]

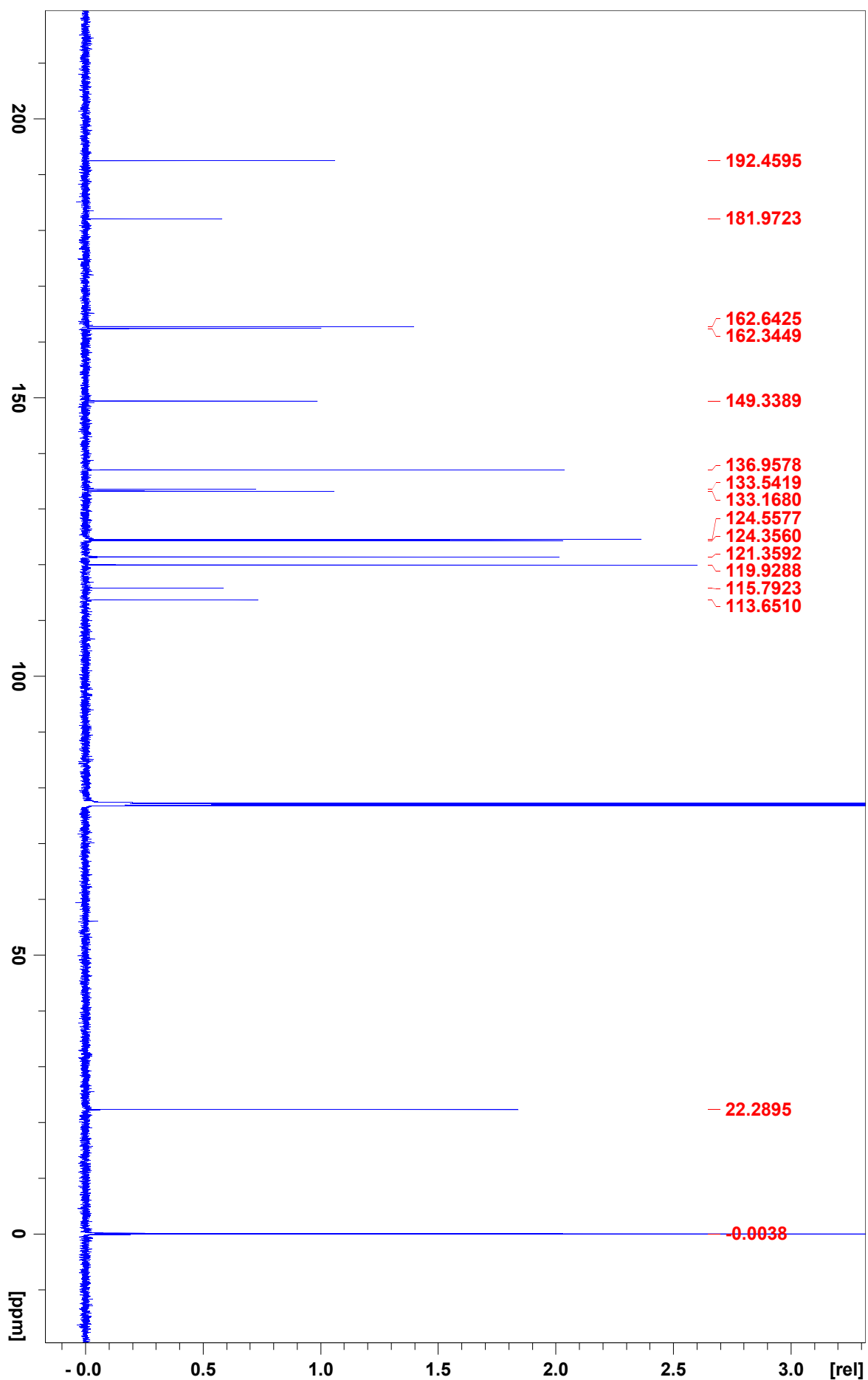

Supplement: Supplementary file 1 [file viruses-15-00903-s001.zip › chrysophanol(13C).pdf]

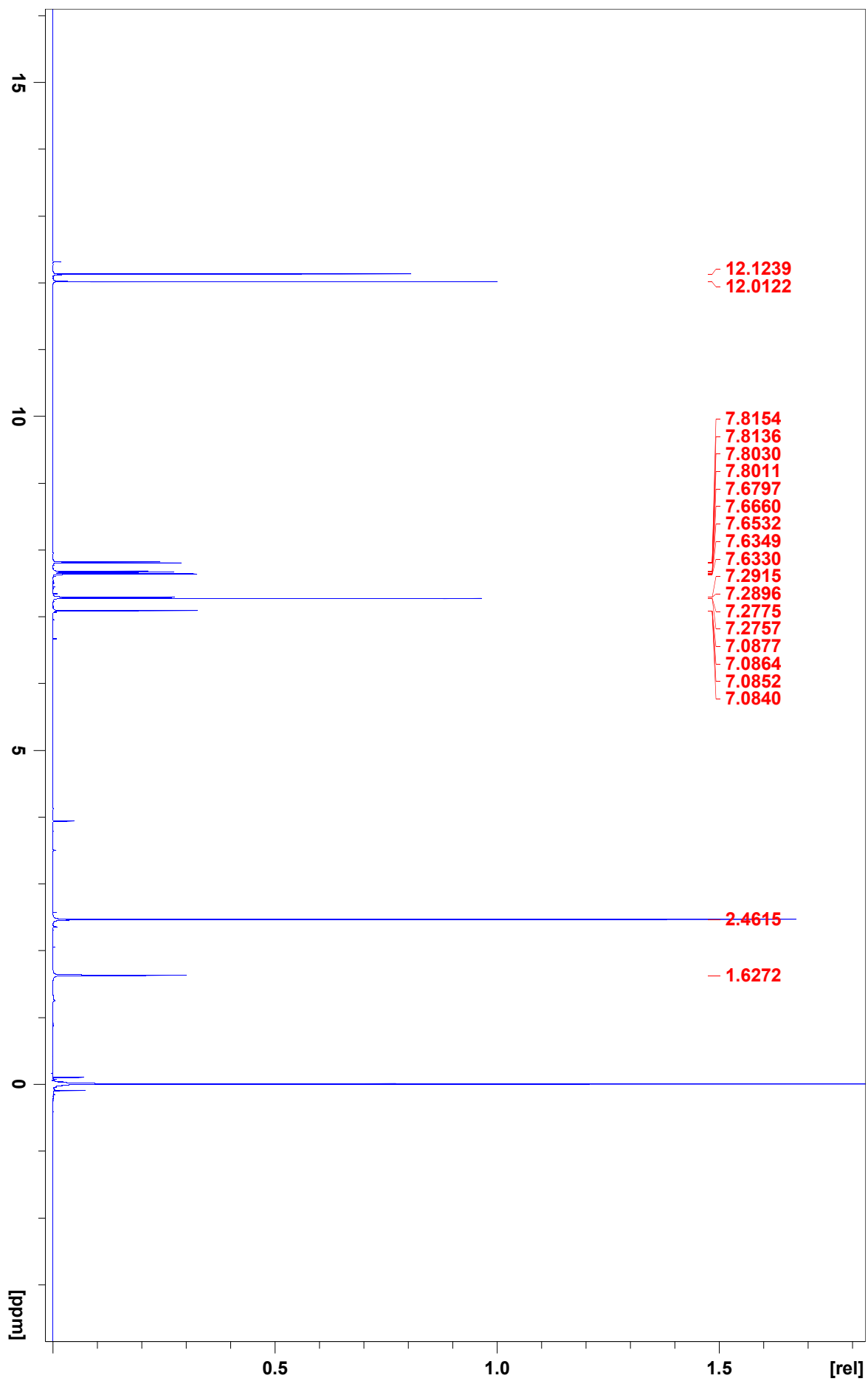

Supplement: Supplementary file 1 [file viruses-15-00903-s001.zip › chrysophanol(1H).pdf]

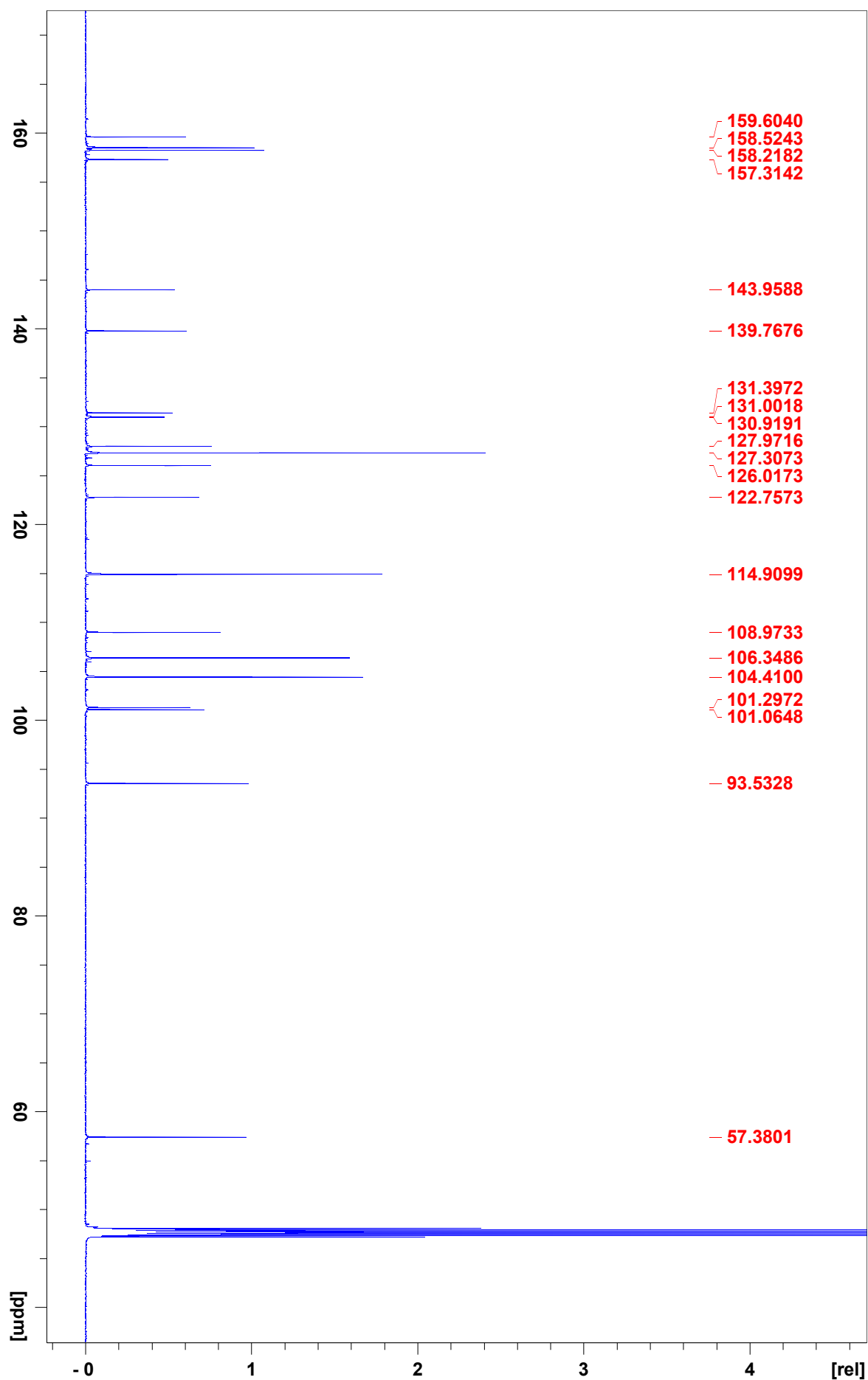

Supplement: Supplementary file 1 [file viruses-15-00903-s001.zip › d-viniferin(13C).pdf]

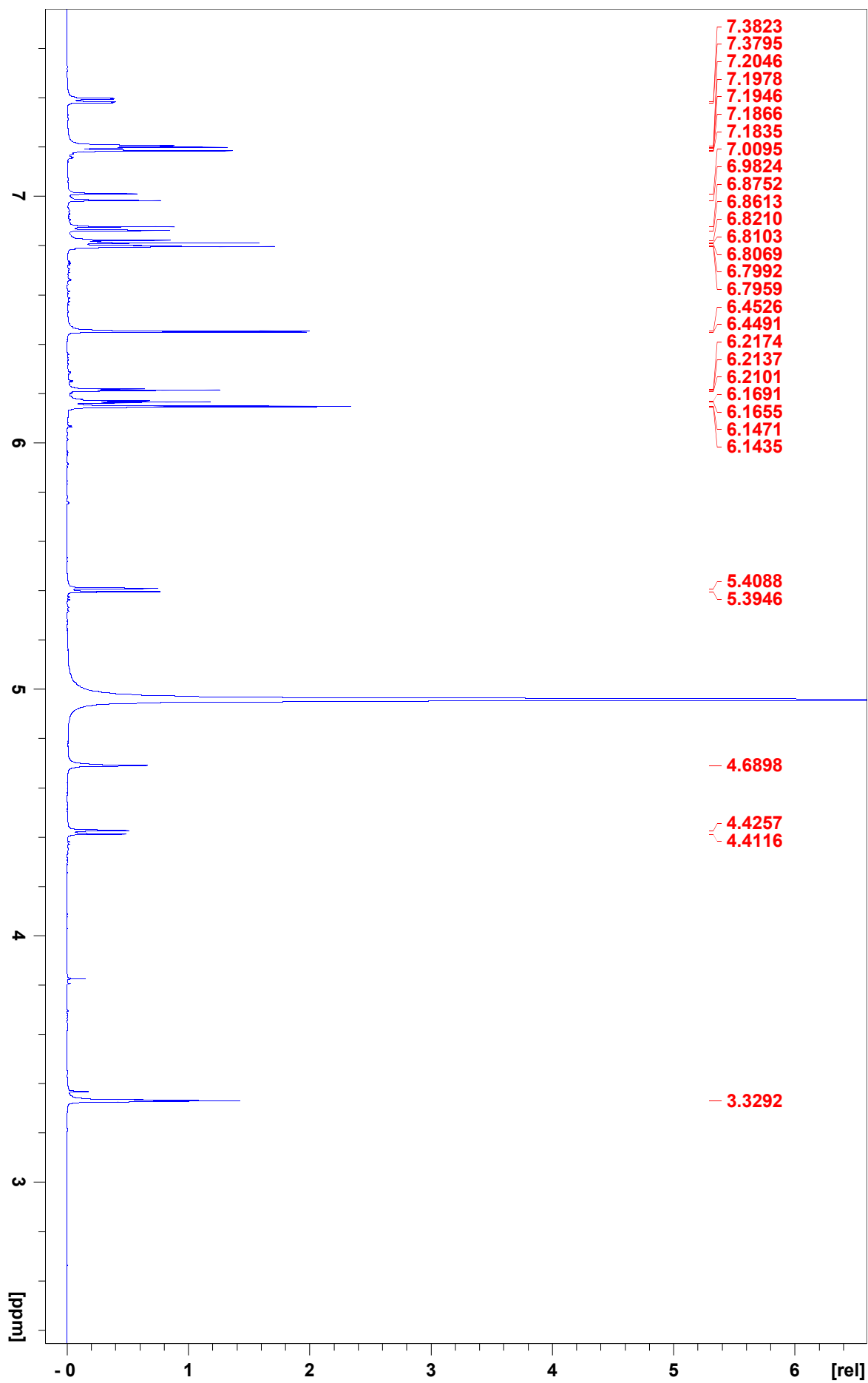

Supplement: Supplementary file 1 [file viruses-15-00903-s001.zip › d-viniferin(1H).pdf]

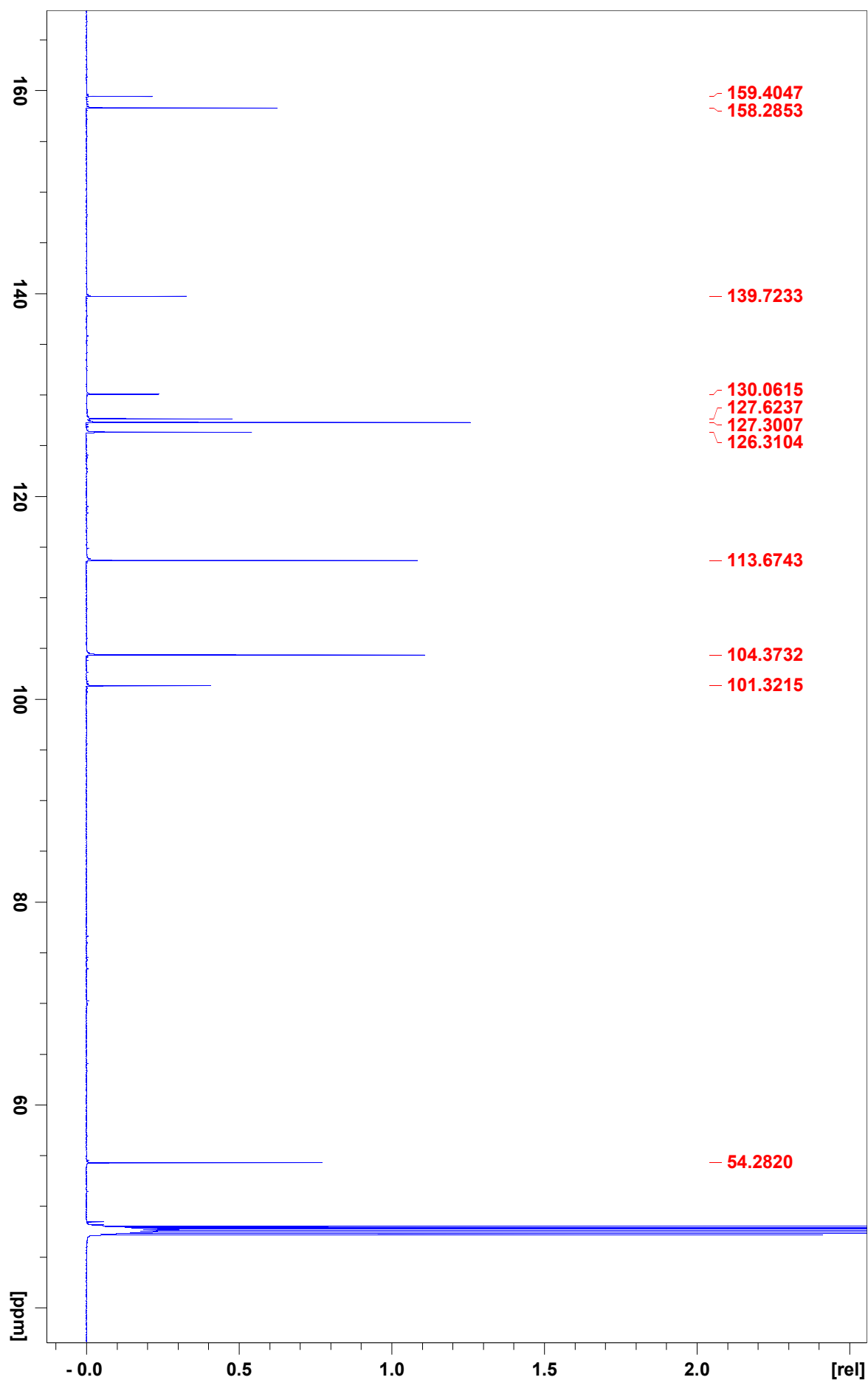

Supplement: Supplementary file 1 [file viruses-15-00903-s001.zip › deoxyrhapontigenin(13C).pdf]

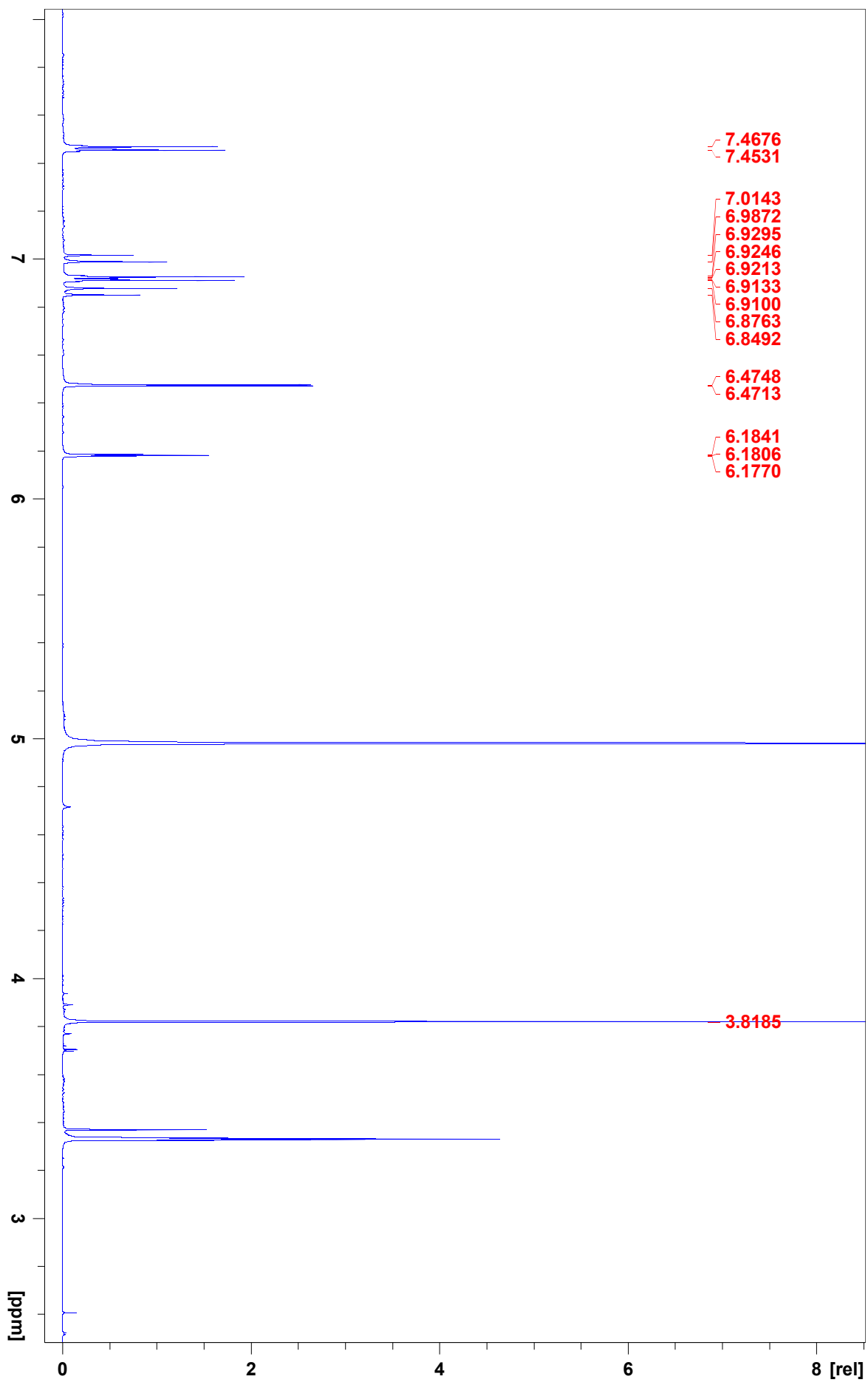

Supplement: Supplementary file 1 [file viruses-15-00903-s001.zip › deoxyrhapontigenin(1H).pdf]

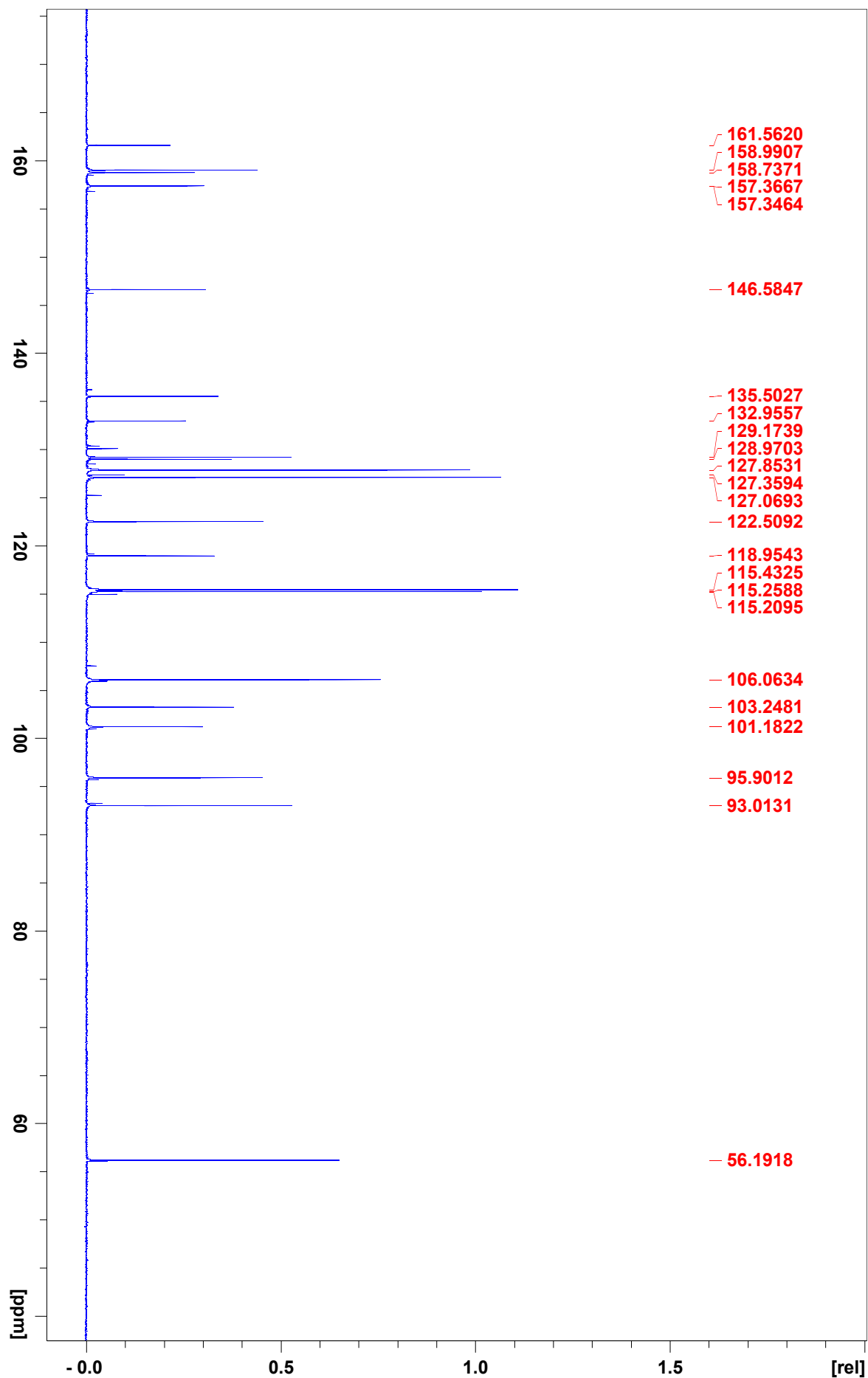

Supplement: Supplementary file 1 [file viruses-15-00903-s001.zip › e-viniferin(13C).pdf]

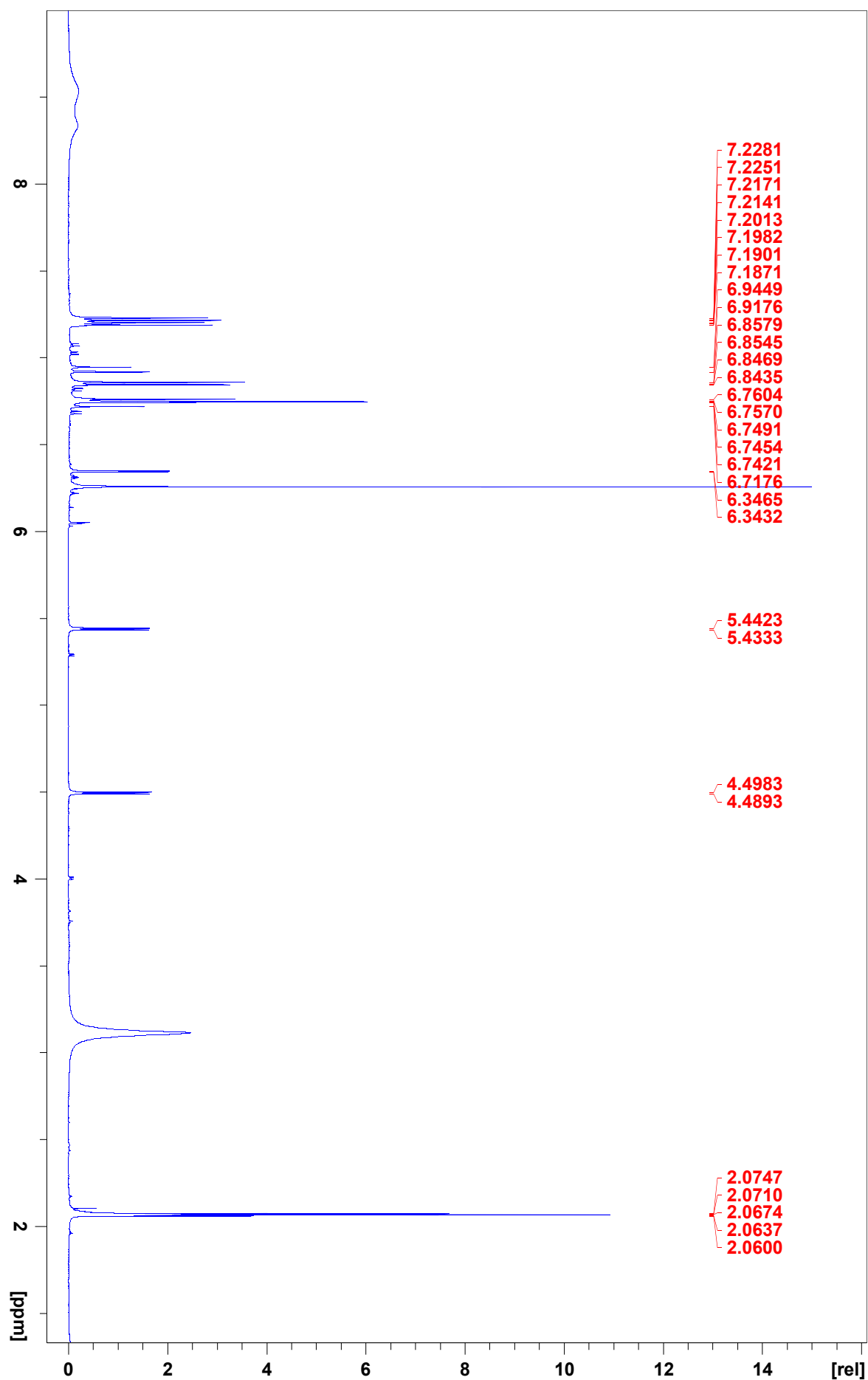

Supplement: Supplementary file 1 [file viruses-15-00903-s001.zip › e-viniferin(1H).pdf]

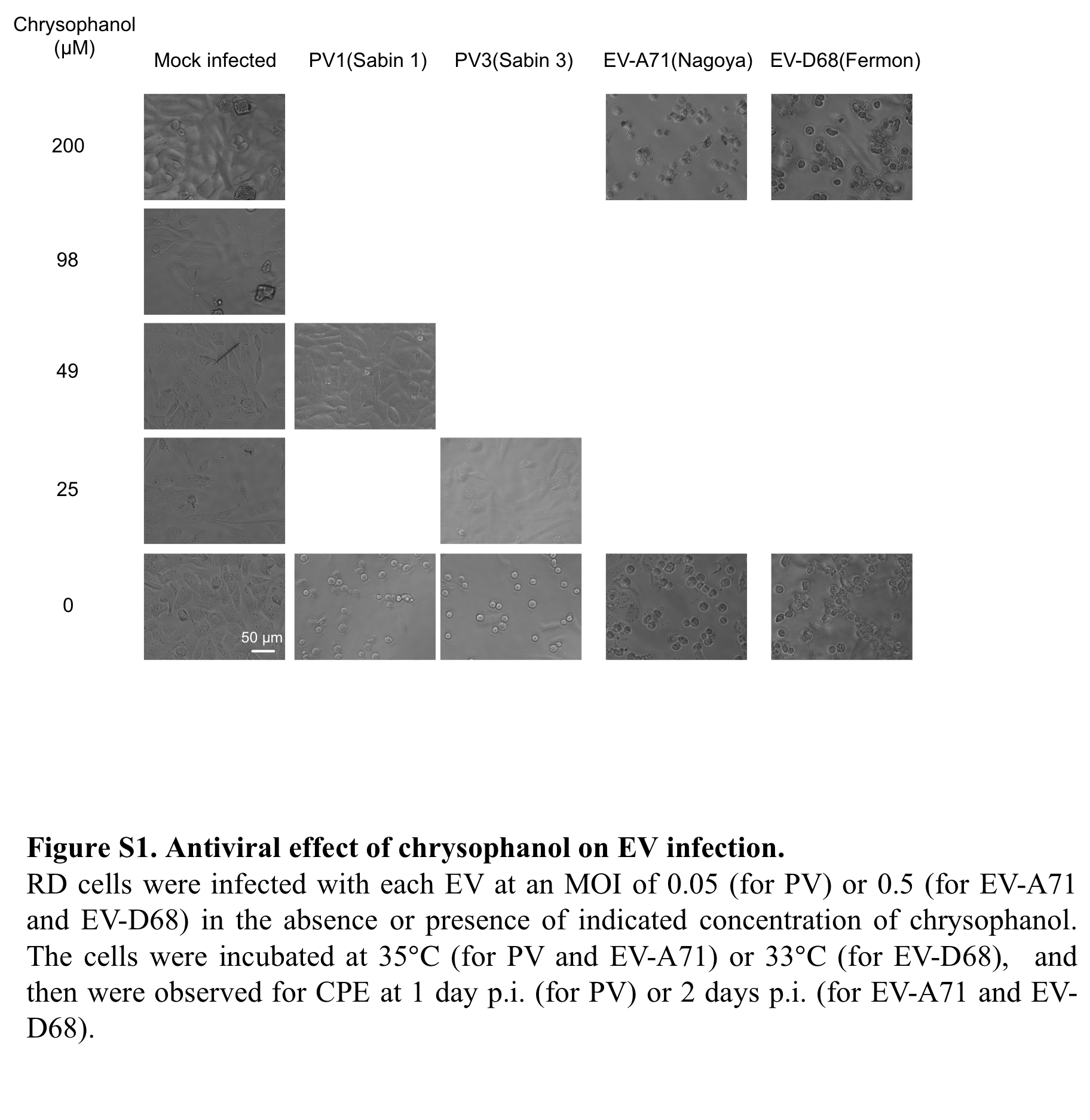

Supplement: Supplementary file 1 [file viruses-15-00903-s001.zip › Figure S1.tiff]

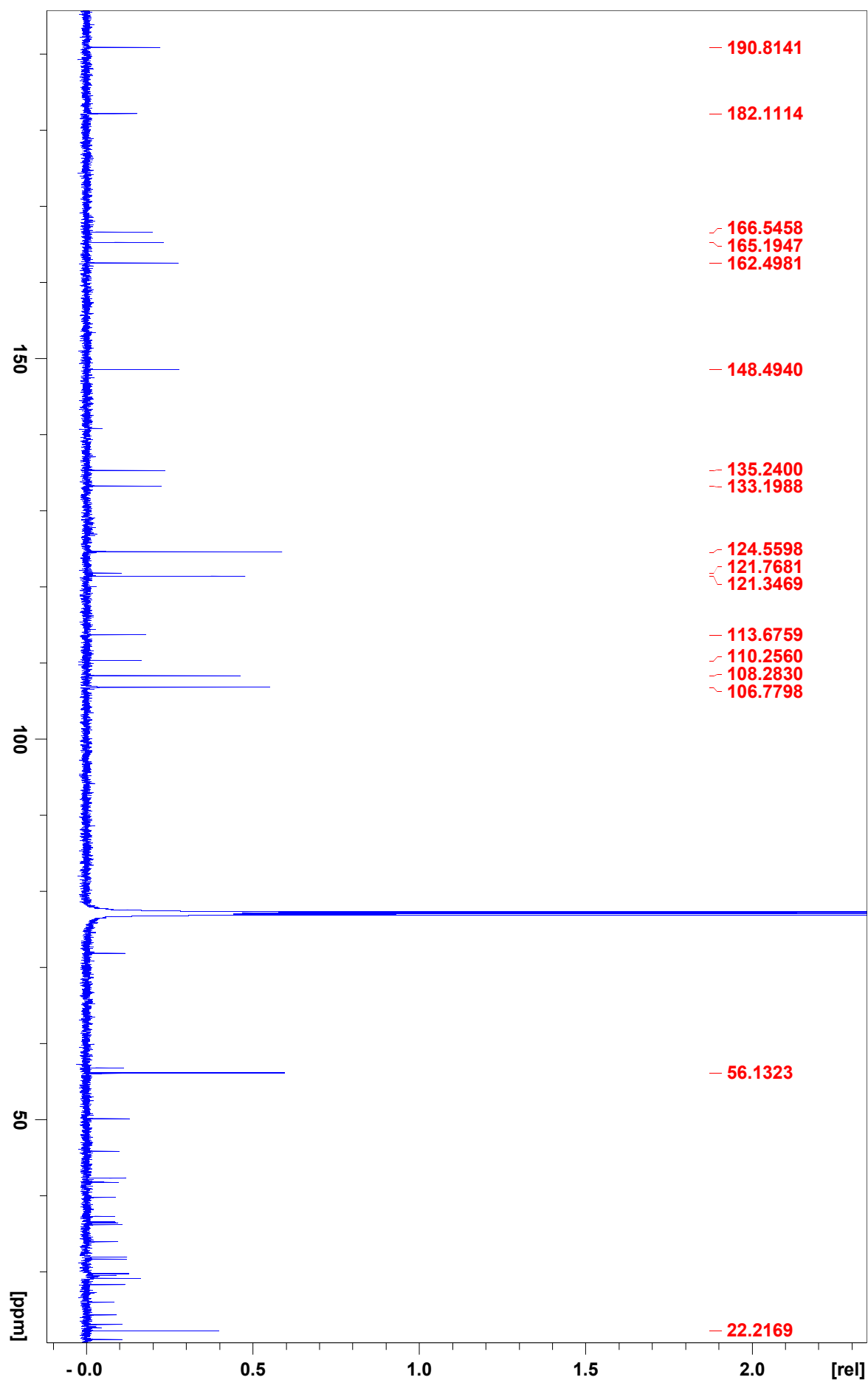

Supplement: Supplementary file 1 [file viruses-15-00903-s001.zip › physcion(13C).pdf]

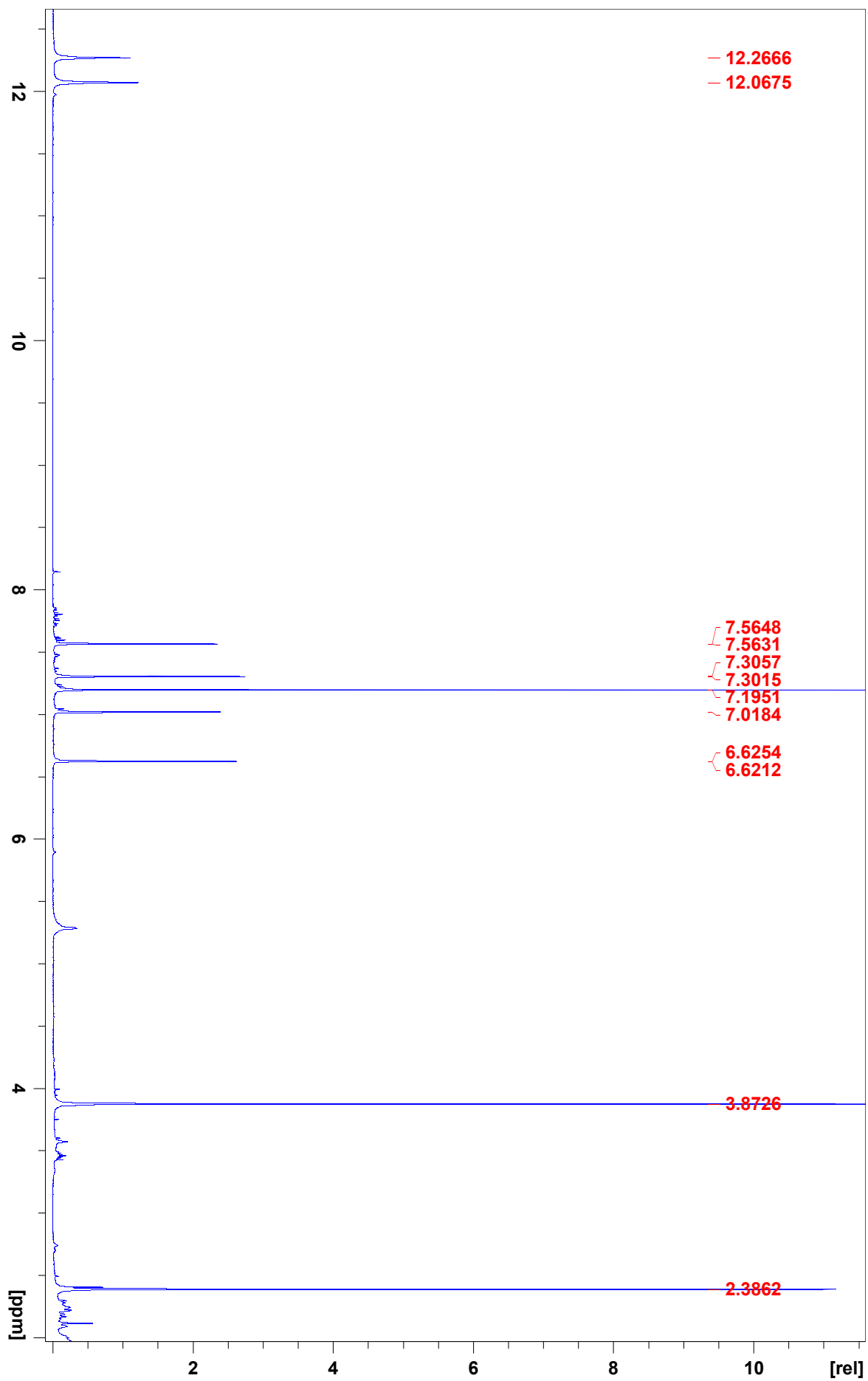

Supplement: Supplementary file 1 [file viruses-15-00903-s001.zip › physcion(1H).pdf]

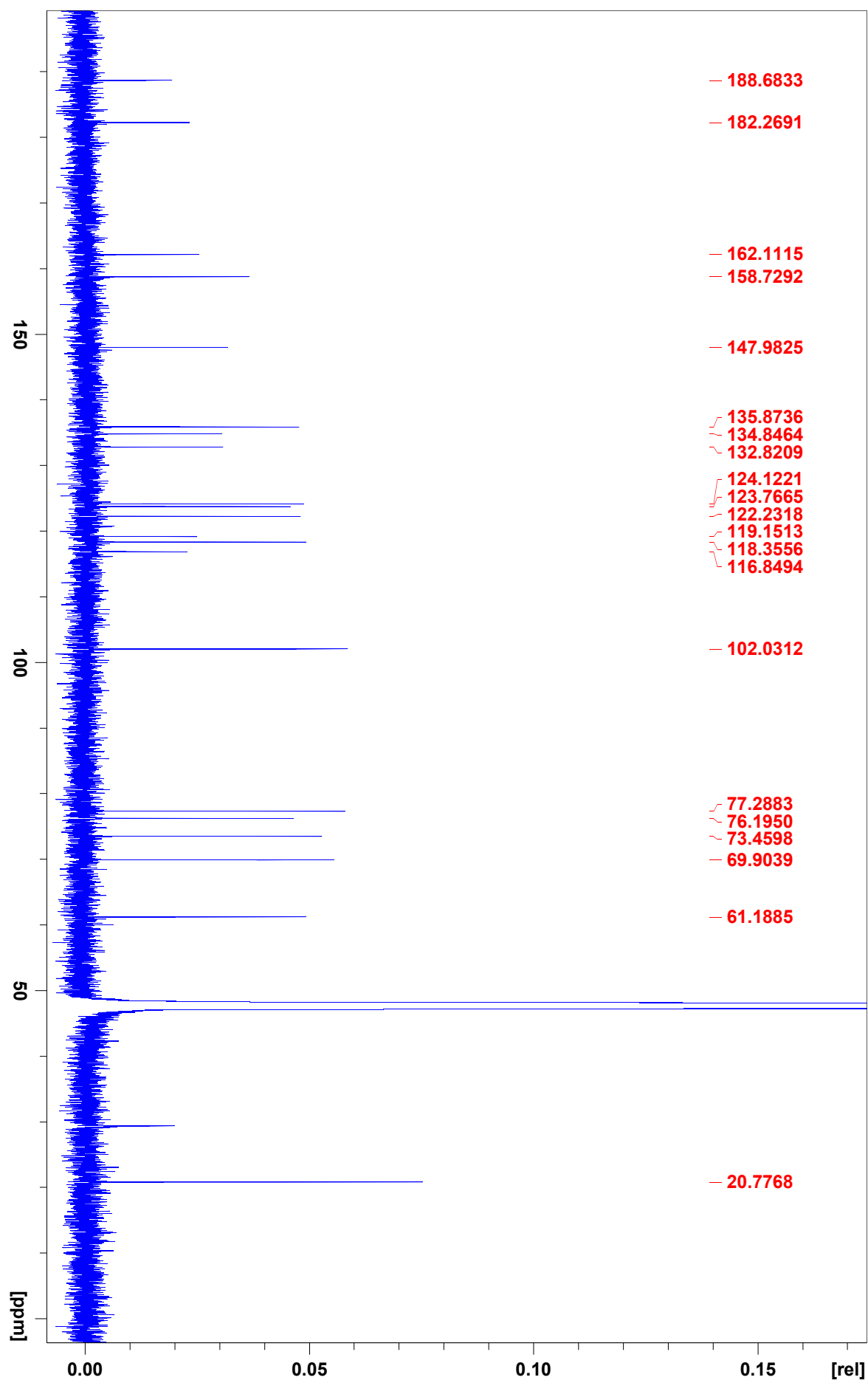

Supplement: Supplementary file 1 [file viruses-15-00903-s001.zip › pulmatin(13C).pdf]

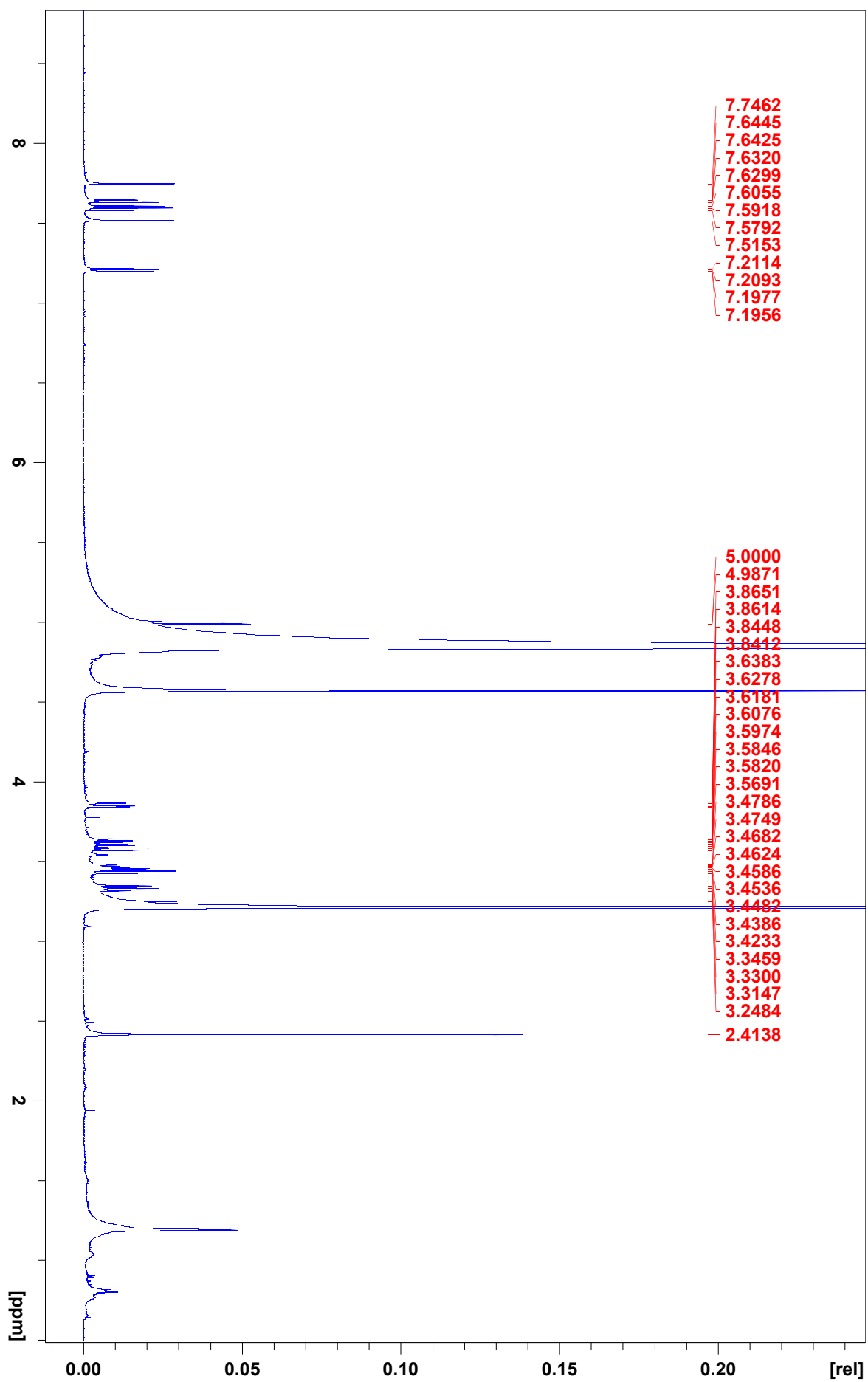

Supplement: Supplementary file 1 [file viruses-15-00903-s001.zip › pulmatin(1H).pdf]

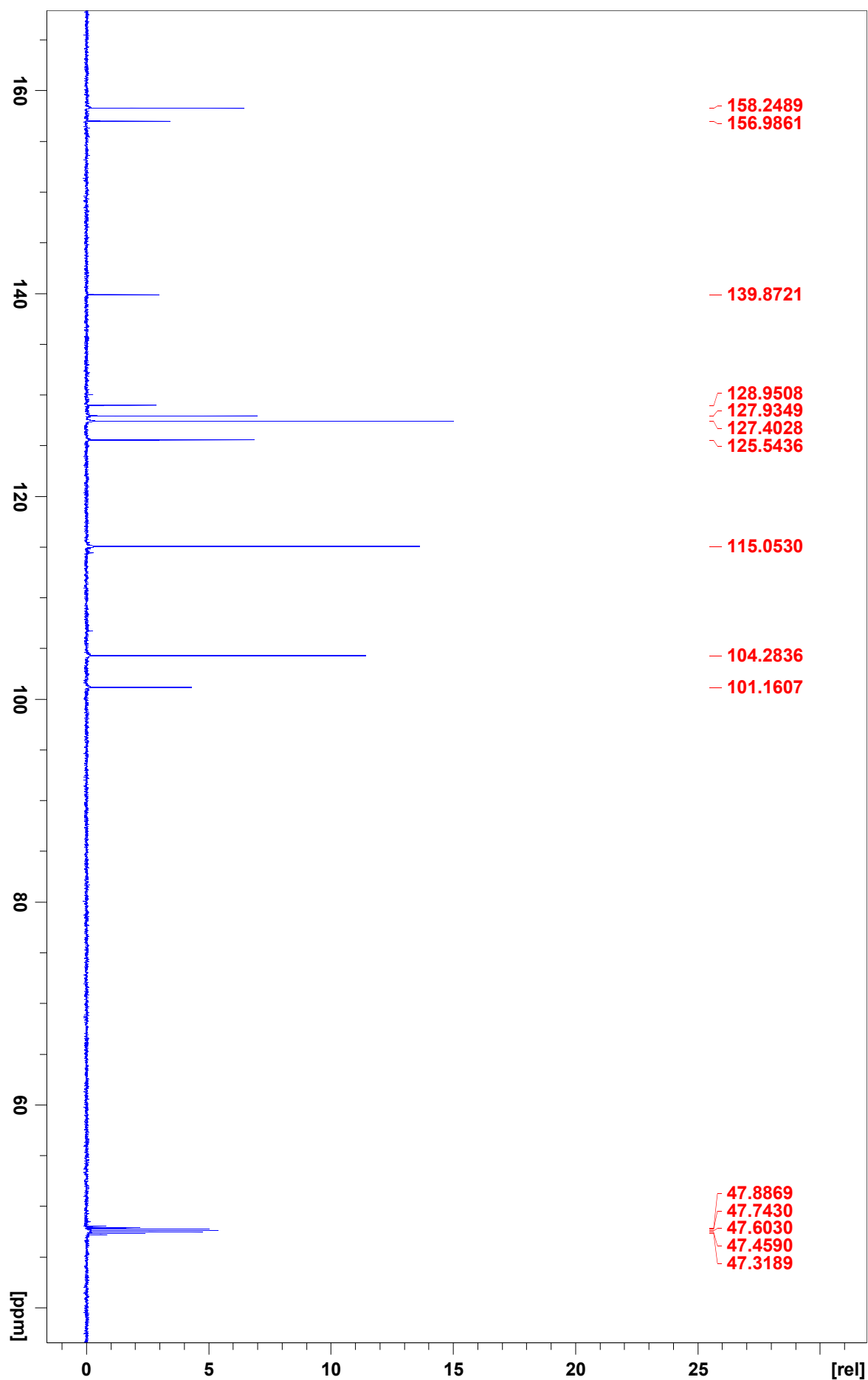

Supplement: Supplementary file 1 [file viruses-15-00903-s001.zip › resveratrol(13C).pdf]

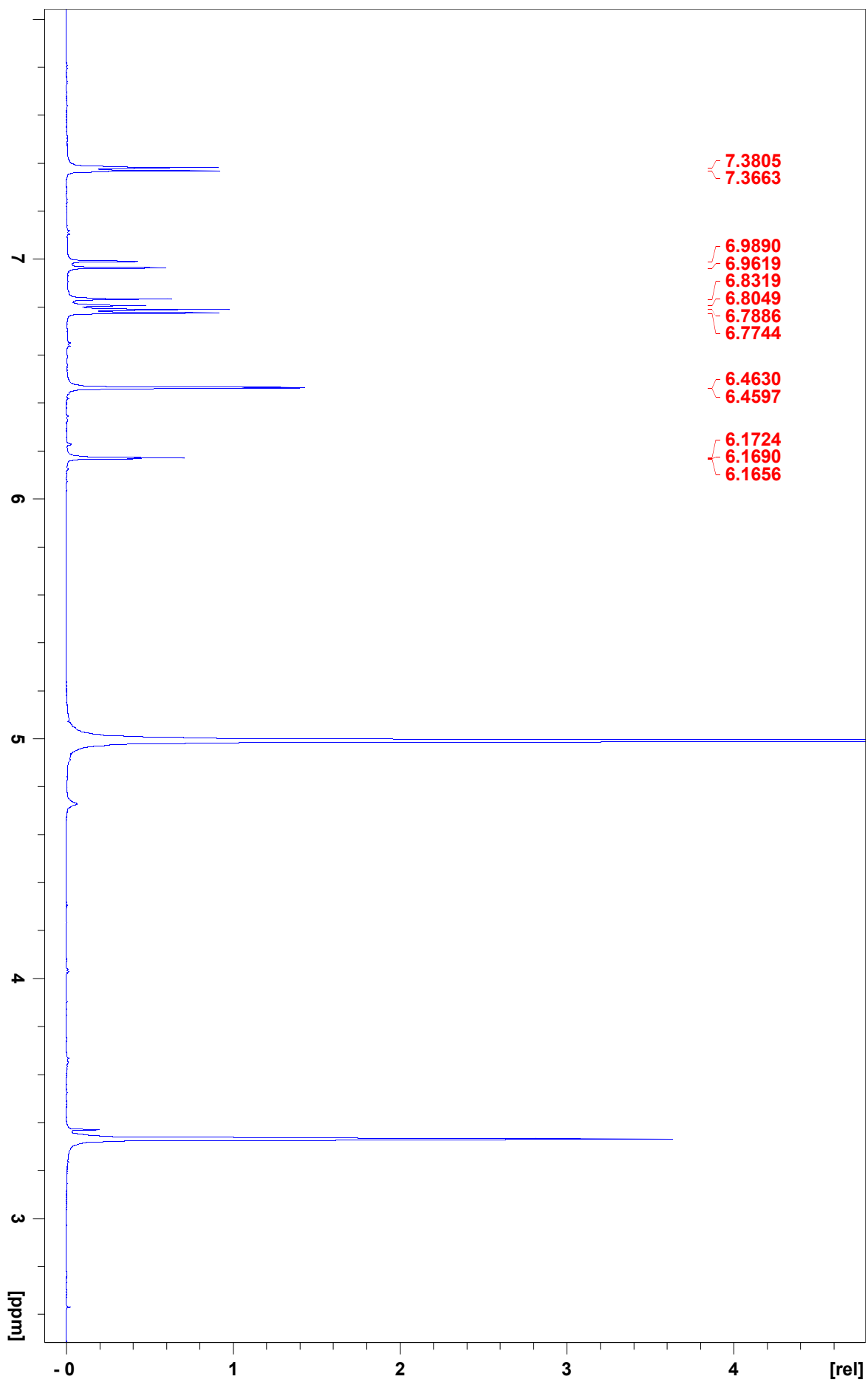

Supplement: Supplementary file 1 [file viruses-15-00903-s001.zip › resveratrol(1H).pdf]

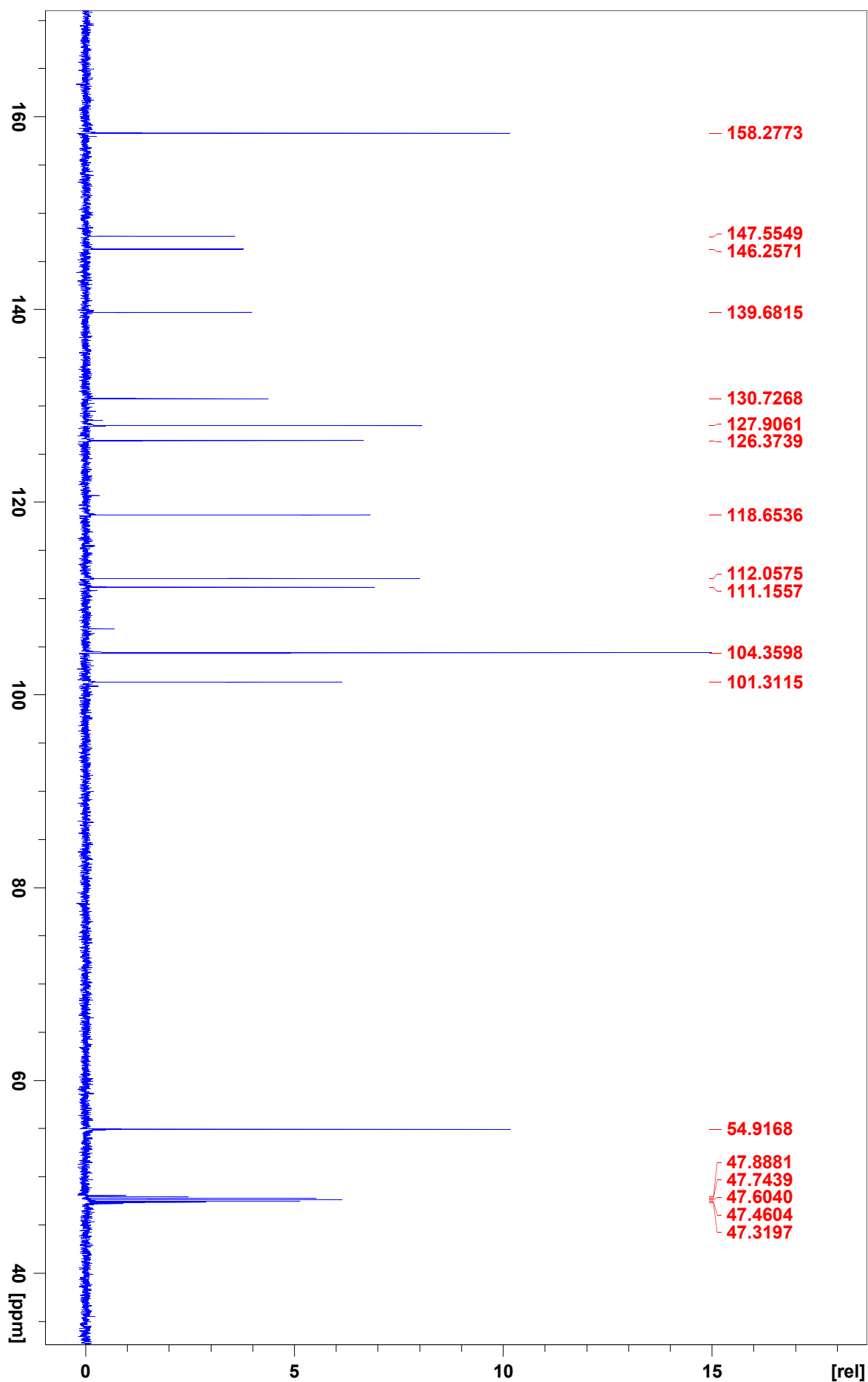

Supplement: Supplementary file 1 [file viruses-15-00903-s001.zip › rhapontigenin(13C).pdf]

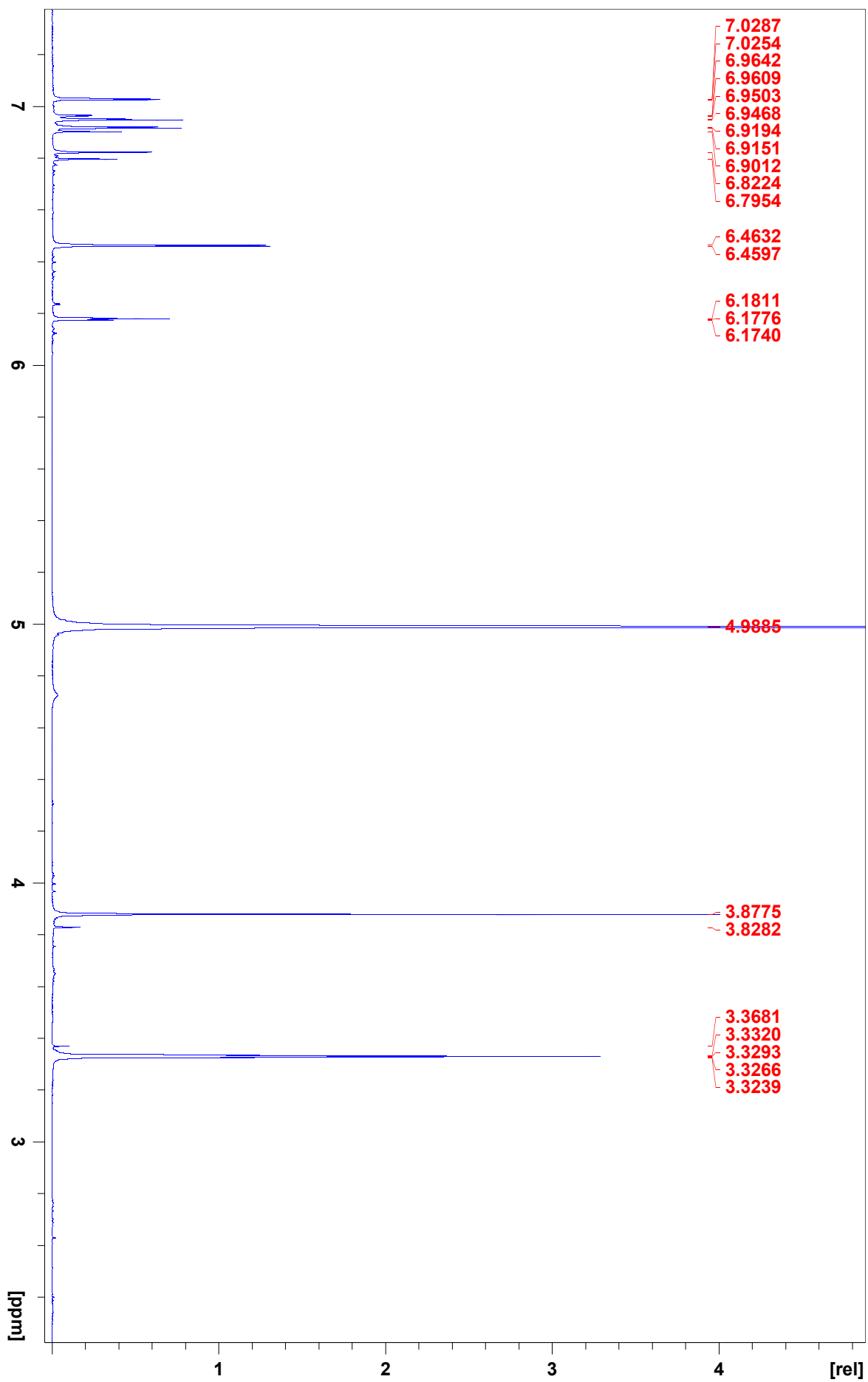

Supplement: Supplementary file 1 [file viruses-15-00903-s001.zip › rhapontigenin(1H).pdf]
